# Eliciting a value set for the Swedish Capability-Adjusted Life Years instrument (CALY-SWE)

**DOI:** 10.1007/s11136-023-03507-w

**Published:** 2023-09-11

**Authors:** Kaspar Walter Meili, Brendan Mulhern, Richard Ssegonja, Fredrik Norström, Inna Feldman, Anna Månsdotter, Jan Hjelte, Lars Lindholm

**Affiliations:** 1 Department of Epidemiology and Global Health, Umeå University, Umeå, Sweden; 2 Centre for Health Economics Research and Evaluation, University of Technology Sidney, Ultimo, Australia; 3 Department of Public Health and Caring Sciences, Uppsala University, Uppsala, Sweden; 4 Respiratory, Allergy and Sleep Medicine Research Unit, Department of Medical Sciences, Uppsala University, Uppsala, Sweden; 5 Department of Social Work, Umeå University, Umeå, Sweden

**Keywords:** Quality-adjusted life year, Time trade-off, Discrete choice experiment, Capability approach, Hybrid modeling, Economic evaluation

## Abstract

**Purpose:**

Our aim was to elicit a value set for Capability-Adjusted Life Years Sweden (CALY-SWE); a capability-grounded quality of life instrument intended for use in economic evaluations of social interventions with broad consequences beyond health.

**Methods:**

Building on methods commonly used in the quality-adjusted life years EQ-5D context, we collected time-trade off (TTO) and discrete choice experiment (DCE) data through an online survey from a general population sample of 1697 Swedish participants. We assessed data quality using a score based on the severity of inconsistencies. For generating the value set, we compared different model features, including hybrid modeling of DCE and TTO versus TTO data only, censoring of TTO answers, varying intercept, and accommodating for heteroskedasticity. We also assessed the models’ DCE logit fidelity to measure agreement with potentially less-biased DCE data. To anchor the best capability state to 1 on the 0 to 1 scale, we included a multiplicative scaling factor.

**Results:**

We excluded 20% of the TTO answers of participants with the largest inconsistencies to improve data quality. A hybrid model with an anchor scale and censoring was chosen to generate the value set; models with heteroskedasticity considerations or individually varying intercepts did not offer substantial improvement. The lowest capability weight was 0.114. Health, social relations, and finance and housing attributes contributed the largest capability gains, followed by occupation, security, and political and civil rights.

**Conclusion:**

We elicited a value set for CALY-SWE for use in economic evaluations of interventions with broad social consequences.

**Supplementary Information:**

The online version contains supplementary material available at 10.1007/s11136-023-03507-w.

## Plain English summary

The Capability-Adjusted Life Years Sweden instrument (CALY-SWE) is a new instrument for measuring quality of life in terms of the freedoms and opportunities of individuals. Its purpose is to be used in cost-effectiveness evaluations for social policies with broad effects, for example, social welfare measures that could affect one’s financial situation and health. For that it is necessary to calculate a rating score from 0 to 1 for all life situations that the instrument describes and that can be used as a quality weight for the time spent in this situation. We asked a Swedish sample of 1697 participants two complementary types of questions in an online survey. The first type were discrete choice experiment (DCE) questions that compared two life situations. The second type were time trade-off (TTO) questions that evaluated how much time in the best situation is equivalent to a longer period in a worse situation. TTO questions can be challenging to understand and to answer, especially online. That is why we excluded TTO data from the participants with the poorest TTO answers to improve the data quality. We then combined DCE and TTO data in a suitable statistical model to derive the rating scores. The health, social relations, and finance and housing attributes were rated as the most important. With the resulting quality weights, it is now possible to conduct economic cost-effectiveness evaluations of quality-of-life policies using CALY-SWE.

## Introduction

Cost-utility evaluations that measure health-related quality of life (QoL) using quality-adjusted life years (QALYs) have become commonplace in areas ranging from mandatory health technology assessments [1, 2] to evaluations of health aspects of social welfare interventions [3]. Crucially, they allow to compare different health interventions in terms of their health effects. QALYs also give intrinsic, explicit value to health as an outcome instead of valuing consequences using money [4]. Considerable methodological expertise around QALYs has accumulated, including valuation methods of preferences for health in the form of standard gamble, visual analogue scale (VAS), and time trade-off (TTO) [4].

However, health-focused QALY instruments such as the EQ-5D instruments [5, 6] or SF-6D [7, 8] are arguably less relevant for principal consequences beyond health, such as social relations or financial issues. Therefore, decision-makers concerned with these areas lack tools for economic evaluations comparable to those in health. In Sweden, for example, municipalities in practice often rely on evaluations that consider costs and savings without attributing intrinsic value to QoL [9, 10]. Consequently, the resulting resource distribution may lack transparency and allocative efficiency compared to more evolved approaches in health care.

To address these issues, we developed the Capability-Adjusted Life Years Sweden (CALY-SWE) instrument targeted at economic evaluations of social interventions, such as preventing high school drop-outs or improving conditions for people with disabilities [11, 12]. While it uses methods and concepts from the QALY context, the instruments’ focus extends beyond health and is based on Amartya Sen’s capability approach [13].

Additionally, measuring distributions of capability-related QoL by CALY-SWE in the population and in subgroups may be informative by itself, outside an evaluation context [14]. Concerns for equality are policy-relevant in Sweden [15, 16] and globally [17].

In light of the need for broader QoL measurement, several instruments have been developed [18, 19], including QALY instruments that consider social aspects [20]. For example, ASCOT [21] focuses on social care, EQ-HWB [22] is a broader QALY instrument for cross-sectoral use, and ICECAP-A [23] is a UK-oriented capability instrument aimed at economic evaluations. In comparison, CALY-SWE focuses on the Swedish context, incorporates equity considerations, and focuses on policy-relevant capabilities [12].

The CALY-SWE attributes were selected by a Delphi process with not-for-profit stakeholders from the Swedish civil society [12], in line with the capability approach that emphasizes context-specificity [24]. The six attributes are health, social relations, financial situation and housing, occupation, security, and political and civil rights (with the three response levels *Completely agree*, *Partially agree*, *Not agree*, See supplementary Table S1).

A necessary component for use in cost-effectiveness evaluations is a set of capability weights for the 729 possible situations that CALY-SWE describes, called *states*. To calculate adjusted life years, the weights should be situated on the [0, 1] scale. For example, a weight of 0.5 for 10 years implies 5 capability-adjusted life-years. For CALY-SWE, we define a weight of 1 to correspond to the capability sufficient for a *flourishing* life [12, 25] and a weight of 0 to 0 lifetime.

No value set has been developed yet for CALY-SWE, but this is required for the use of CALY-SWE in economic evaluations.

### Aim

Our aim was to elicit a value set for the CALY-SWE instrument with two purposes: (1) for use in economic evaluations and (2) for describing CALYs in the Swedish population. This study constitutes a key step in the development of CALY-SWE.

## Methods

### Overview

Given the conceptual inspiration of the CALY-SWE instrument in health-economic cost-utility analysis, we chose to rely on methodology widely used for value sets, namely TTO and DCE tasks [4, 26]. TTO has since its inception been considered a simpler alternative to standard gamble [4, 27], and DCEs based on random utility theory have a long history in choice behavior modeling [28].

TTO questions in the survey contained a choice between two hypothetical life courses: (1) to live 10 years in an imperfect capability state or (2) to live a period from 1 to 10 years in the full capability state with health, social relations, financial situation and housing, occupation, security, and political and civil rights all on level 3 (denoted as 333333, in listed order, with levels from 1—*Do not agree* to 3—*Completely agree*). Depending on the choice, the number of years with full capability was adjusted iteratively until participants reached an indifference point of *x* years (Supplementary section *Iteration procedure*). The TTO weight is given by $w=x/10$ because $x*1=w*10$ (*1* is the weight for 333333, *10* is the number of years with imperfect capabilities, and *x* is the TTO answer). In the DCE questions participants picked one of two hypothetical states (Supplementary section *Survey screenshots*).

TTO and DCE provide complementary information with different properties [4]. The TTO question format uses time as a reference and measures the absolute value of single states on the [0, 1] scale. The TTO iteration procedure may be cognitively challenging and thus introduce bias [29, 30], as does the expectation of linear time preferences [31]. In DCE questions, participants compare two distinct states without a reference point resulting in information on the relative strengths of attributes and levels. While DCE questions may be easier to understand [32], the results are not located on the desired [0, 1] scale [33]. Combining the two measures offers potentially less biased DCE data with TTO anchoring and the possibility to model the value of capabilities with preference information from two different angles.

This approach largely draws upon proven methodology developed for EuroQol’s EQ-5D-3L and EQ-5D-5L instruments for which numerous country-specific value sets exist [5, 34–40].

### Survey and experimental design

Additional details are available elsewhere, including the survey development [41]. We constructed the survey using the scripting language PHP and the template engine twig [42, 43]. It contained the following sections: (1) informed consent, (2) the CALY-SWE instrument for self-completion and a VAS question [44], (3) the DCE block including 6 DCE tasks, (4) the TTO block including 5 TTO tasks, and (5) background questions.

We chose unsupervised, self-administrated online administration because, (1) interviewer renumeration, recruiting, and training is resource-intensive, (2) uncertainty regarding the feasibility of physical meetings linked to Covid-19, (3) participants could directly be sampled according to representative quotas and redirected to the survey, and no scheduling was required for matching with an interviewer. Participants were sampled via the panel company CINT [45] with representative quotas for Sweden for gender, region, and age, from January 3 to April 18 2022. To assess representativeness, we compared self-reported data on gender, birthplace, education, age, income, and municipality size with data from Statistics Sweden (SCB) on population, education, and household finances [46–48] using Chi-squared tests. For income we graphically compared probability masses because the survey income answer categories were not directly comparable to those used by SCB.

For the TTO questions, we generated a D-optimal design using the skpr package [49] (D-efficiency 85.83%). We generated a design with eight blocks and three states each, totalling 24 states. We augmented each block with the pit state 111111 and a *learning* state with three attributes on level 2 and three on level 3. Thus, the learning state dominated at least one other state in the block besides 111111. The learning state was displayed first, and the order of the remaining TTO states was randomized. The learning state guided participants through the first two iterations with pre-determined choices accompanied by explanations, restricting answers to [0.2, 0.9] compared to [0.1, 1] for the other states.

Based on the orthogonal array approach outlined in Street et al. [50], we developed a D-optimal DCE experimental design with 43 choice sets (D-efficiency 100%) and displayed five randomly selected choice sets in random order per participant. As a consistency check, we added the dominated choice between 222332 and 232332 at a random position.

### Sample size

We determined the sample size using a simulation with a hybrid model [41, 51], with parameters informed by earlier pilot data. We assessed the mean absolute error (MAE) and 95% credible interval (CI) widths of recovered weights and arrived at minimum sample sizes of 500 and 1000 participants for a hybrid model and a TTO-only model, respectively. To leave a safety margin, we aimed for 1500 participants. Finally included were around 1700 participants, including 200 participants from the initial stage, resulting in approximately 210 valuations per TTO state and 200 per DCE pair.

### Data quality and TTO and DCE data characteristics

In valuation studies with the aim of generating value sets, considering data quality is important. Possible reasons for low-quality data include confusion about the task or a lack of engagement [52]. Respondents receiving incentives potentially speed through the survey, stating inconsistent responses [53, 54].

We used the concept of inconsistency for data quality assessment. A dominated pair occurred if at least one level of a state ${s}_{1}$ was higher than for another state ${s}_{2}$ while the other levels were equal. A *weak* or *strict* inconsistency occurred for corresponding TTO answers ${w}_{1}$ and ${w}_{2}$ if ${w}_{1}\le {w}_{2}$ or ${w}_{1}<{w}_{2}$, respectively. We calculated the *combined inconsistency severity* (CIS) score to reflect the severity of the weak inconsistencies per-participant (Supplementary sections *CIS score* and *Inconsistencies*). We analyzed the impact of excluding data according to CIS on model fit (details reported elsewhere [41]) and of excluding data on the representativeness by comparing the characteristics of all and the excluded participants. We did not exclude DCE data.

As a basic validity test, we examined in a scatterplot if the mean TTO answer per state showed a positive relation with the level sum score (LSS) per state; with the LSS being a proxy for states’ QoL [34, 55]. Similarly, for DCE, we assessed the LSS differences between the two states plotted against the choice proportions, expecting a pattern of higher choice proportions for higher differences in LSS [34].

### Modeling and anchoring

For all data analyses, we used the statistical software R [55]*.* We implemented the models in a Bayesian framework using Stan [56] with the cmdstanr R interface [57]. The basic model comprised 12 additive coefficients plus intercept. Coefficients for level 2 encoded the difference to the constant, and coefficients for level 3 encoded the difference to level 2 and both were restricted to be positive:

$${y}_{i}\sim\upmu +{X}_{i}\upbeta +{\upepsilon }_{i},\left(\upmu ,\upbeta \ge 0\right)$$

*μ*: constant, *β*: coefficients for the attribute levels.

We adopted the hybrid model presented by Ramos-Goñi et al. [51] with a linear regression component for TTO and a logit regression component for DCE with a multiplicative scaling factor applied to the logit coefficients. Stan example code and the coding scheme are provided in the Supplementary section *Model specification*.

Restricting TTO answers between 1 and 10 years commonly results in a skewed distribution towards these censor points, which is at odds with normally distributed errors [58, 59]. Consequently, we explored TTO models with left-censoring at 0.1 and right-censoring at 1 or at 0.2 and 0.9, respectively, for the learning state.

Heteroscedasticity, where the variance is not constant across the answer range, is common in TTO data [34, 35, 60, 61]. We used the Breusch-Pagan test [62, 63] to assess heteroscedasticity and attempted to capture it by modeling the standard deviation’s logarithm with the same parametrization as the main linear TTO outcome [35]:

$$log\left(s{{d}_{\upepsilon }}_{i}\right)\sim {\upmu }_{H}+{X}_{i}{\upbeta }_{H}+{{\upepsilon }_{H}}_{i},{\upbeta }_{H}\ge 0$$

The TTO task implies that 333333 has a weight of 1 (full capability), an anchoring that has been widely adopted for preference-based instruments for health-economic evaluations [8, 64–66]. However, the weight predicted for 333333 by the basic TTO model does not necessarily equal 1 but represents the extension of the fitted linear model. To deal with this, we tried two approaches: First, we used a coding scheme for the TTO linear regression where coefficients correspond to *discapability* and removed the constant, implying that 333333 is equal to 1:

$$1-{y}_{i}\sim {X}_{i}\beta +{\epsilon }_{i},\left(\beta \ge 0\right)$$

Second, we introduced an *anchor scale* for the TTO linear regression into the model and restricted the weight for 333333 to 1 with a very strong prior standard deviation (0.01) to ensure sufficient precision. Applying the anchor scale only to the level attribute coefficients does not affect the constant, thus anchoring the weights relative to the pit state and 1:

$${y}_{i}\sim\upmu +{X}_{i}\upbeta \cdot s+{\upepsilon }_{i}$$

$$1\sim\upmu +{\overrightarrow{x}}_{333333}\upbeta +{\upepsilon }_{i}$$

*s*: anchor scale factor, $\overrightarrow{x}$: is the model vector for 333333.

We refer to models where the TTO outcome variable is encoded as $y=1-x$ as using the *discapability* specification and to models where $y=x$ as using the *attainment* specification.

### Model comparison

To compare coefficients resulting from TTO data with preferences derived from the potentially less biased DCE questions, we calculated a *logit fidelity* score that corresponded to the sum of absolute differences between logit DCE and comparator model coefficients without constant. Before that, to account for the otherwise incomparable scales, the coefficients were normalized per model so that their sum corresponded to 100% (*β*_*c*_ and *β*_*r*_ corresponding to comparator and reference model coefficients, respectively):

$$\sum_{i}\left|\frac{{B}_{{C}_{i}}}{\sum {B}_{c}}-\frac{{B}_{{r}_{i}}}{\sum {B}_{r}}\right|$$

We also conducted a *kfold* cross validation [67] where we divided the data into 10 equally sized parts and used nine parts for fitting and the remaining part to assess the predictions. This was repeated once for each of the 10 parts. We calculated and averaged the following measures: DCE accuracy equalling to the proportion of correctly predicted DCE choices; and the mean error (ME), MAE, and the mean squared error (MSE) of the predicted TTO answers compared to the observed answers.

We compared a set of models with different features, including TTO or hybrid, anchor scale or discapability specification, with or without varying intercept, and with or without heteroskedasticity. We calculated percentile scores and mean ranks for these models based on the following metrics: kfold DCE accuracy, kfold MAE, distance of 333333 to 1, logit fidelity, and the mean 95% CI of all weights relative to the range covered. To assess the effect of excluding data, we conducted sensitivity analyses for including 50%, 60%, 70%, 80%, 90%, and 100% of the TTO data for the finally selected model.

## Results

### Sample characteristics

Of 8378 invited participants, 2569 (30.7%) accepted to participate, of which 1703 (66.3%) completed the survey. We excluded six participants with a stated age of less than 18, and 53 TTO answers from 37 participants because of technical issues, resulting in a sample of 1697 participants.

Compared to the Swedish population, the sample contained more Swedish-born people; fewer people with less than 9 years of schooling, fewer who only finished elementary schooling, and fewer with a shorter high school education; more people with longer high school and tertiary education; fewer people in municipalities with fewer than 100,000 inhabitants and more people from cities with over 100,000 inhabitants; and finally fewer people in the age group 81–90 years. From the variables used for representative sampling, gender and region did not differ significantly from population proportions, but age did. Visual inspection revealed an income distribution similar to the population income distribution but with higher monthly income proportions between 24,000 and 30,000 SEK (Supplementary Fig S4).

### TTO and DCE answer distributions

**Fig. 1:**
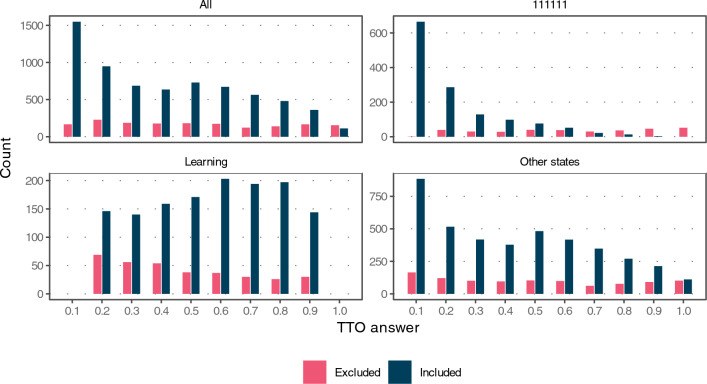
Histograms of the TTO answers for all data (used to generate the value set after exclusions), and stratified by the state 111111, the learning state, and the other states (without 111111 and the learning state). Separate sets of bars represent data that were included or excluded according to the CIS score. *TTO* time trade-off, *CIS* combined inconsistency severity

The TTO answer distribution was accentuated at 0.1, with a total of 25.4% of non-learning states valuated at this value before data exclusion. Clustering around the lowest value occurred especially for the pit state, but also for other states. Answers that valued 111111 high and the learning state low were excluded more frequently (Fig. 1).

**Fig. 2:**
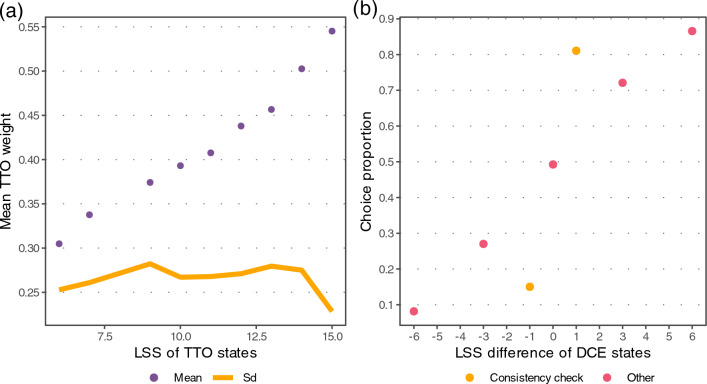
State severity vs. answer distribution. **a** The mean TTO answer and standard deviation (SD) vs. the LSS of the TTO states. **b** Proportion of choosing the first DCE state vs. the LSS difference compared to the second state. TTO time trade-off, *DCE* discrete choice experiment, *LSS* level sum score

The LSS of the TTO states were clearly correlated with the mean answers. In the DCE questions, the choice proportion of the first state was also correlated with the LSS difference to the second state. The standard deviation of the TTO answers was largely constant across the LSS range, except for the highest LSS of 15 where the standard deviation dropped (Fig. 2). Conversely, the Breusch–Pagan test indicated evidence for heteroscedasticity. Additional descriptive timing and TTO answer data are reported in Supplementary Tables S3–S5, Fig. S5, S6).

### Data quality and exclusion

We excluded TTO data from 20% of participants according to the CIS score because their data worsened the TTO linear model fit [41]. The remaining sample’s background characteristics did not differ substantially from the overall sample (Table 1, Supplementary Table S2, Fig. S4, S7).

**Table 1:** Background characteristics

	Sample		Included TTO		Population
Category	*N*	(%)	*N* weighted	(%)	%
Age					
Mean (1st quartile, 3rd quartile)	48.73	(33, 64)	49.44	(33, 64)	
Birthplace (*X*^2^: 212.2***)					
Sweden	1529	(90.47)	1235	(91.97)	77.34
Other Nordic country	33	(1.95)	25.4	(1.89)	2.56
Europe (West)	21	(1.24)	15.8	(1.18)	1.39
Europe (South)	16	(0.95)	13	(0.97)	1.73
Europe (East)	27	(1.6)	17.8	(1.33)	3.81
Africa	8	(0.47)	3	(0.22)	2.52
Western Asia	28	(1.66)	15	(1.12)	5.77
Southern Asia	4	(0.24)	3	(0.22)	1.82
Southeast Asia	4	(0.24)	1	(0.07)	1
Eastern Asia	6	(0.36)	5	(0.37)	0.64
North America	4	(0.24)	3	(0.22)	0.29
Latin America	4	(0.24)	4	(0.3)	1.07
Rest of the world	6	(0.36)	1.8	(0.13)	0.07
Abstain	7		4		
Total	1697	(100)	1347	(100)	100
Region (*X*^2^: 7.472, *df*: 7)					
Stockholm	357	(21.14)	283.6	(21.13)	22.89
East-Central Sweden	286	(16.93)	218.4	(16.27)	16.75
Småland and islands	150	(8.88)	119.4	(8.9)	8.41
South Sweden	251	(14.86)	200.8	(14.96)	14.87
West Sweden	322	(19.06)	264.4	(19.7)	19.95
North-Central Sweden	155	(9.18)	121.4	(9.05)	8.35
Central Norrland	69	(4.09)	51	(3.8)	3.65
Upper Norrland	99	(5.86)	83	(6.18)	5.13
NA	8		5		
Total	1﻿697	(100)	1347	(100)	100
Education (*X*^2^: 169.3***, *df*: 5)					
Less than 9 years schooling	31	(1.83)	22.8	(1.7)	6.5
Finished elementary (9 years)	132	(7.8)	97.2	(7.23)	10.22
High school or vocational (2 years)	225	(13.3)	186.8	(13.9)	20.4
High school (3–4 years)	463	(27.36)	372.8	(27.74)	23.48
Tertiary education (shorter than 3 years)	346	(20.45)	264.2	(19.66)	14.95
Tertiary education (3 years or more)	495	(29.26)	400.2	(29.78)	24.45
Abstain	5		3		
Total	1697	(100)	1347	(100)	100
Gender (*X*^2^: 0.1074, *df*: 1)					
Woman	846	(50.36)	673.2	(50.43)	49.96
Man	834	(49.64)	661.8	(49.57)	50.04
Other	9		8		
Abstain	8		4		
Total	1697	(100)	1347	(100)	100
Housing (MC)					
More or less without housing	17	(1.02)	12	(0.91)	
Renting	708	(42.55)	536.8	(40.73)	
Own an apartment	330	(19.83)	269	(20.41)	
Own a house	588	(35.34)	485.2	(36.81)	
Student room or shared living	21	(1.26)	15	(1.14)	
Other	47		42		
Abstain	18		13.8		
Total	1729	(100)	1374	(100)	
Income					
Less than 24 k SEK	600	(38.54)	487.8	(39.31)	
24 k to 27 k SEK	240	(15.41)	180.2	(14.52)	
27 k to 30 k SEK	206	(13.23)	163.8	(13.2)	
30 k to 50 k SEK	408	(26.2)	324.6	(26.16)	
More than 50 k SEK	103	(6.62)	84.4	(6.8)	
Abstain	140		106.2		
Total	1697	(100)	1347	(100)	
Living situation (MC)					
Single	516	(25.23)	410.6	(25.26)	
With partner	951	(46.5)	747.4	(45.98)	
With parents	97	(4.74)	79.2	(4.87)	
With sibling	36	(1.76)	31.8	(1.96)	
With children (own or other)	445	(21.76)	356.4	(21.93)	
Other	28		21		
Abstain	7		6		
Total	2080	(100)	1652	(100)	
Municipality size (*X*^2^: 172.2***, *df*: 4)					
Less than 20 k inhabitants	288	(17.13)	247.8	(18.55)	21.5
20 to 50 k inhabitants	295	(17.55)	232.4	(17.4)	24.73
50 to 100 k inhabitants	336	(19.99)	258	(19.31)	21.62
100 to 300 k inhabitants	338	(20.11)	280.6	(21)	16.91
Big city (Stockholm, Gothenburg, Malmö) with > 300 k inhabitants	424	(25.22)	317.2	(23.74)	15.24
Abstain	16		11		
Total	1697	(100)	1347	(100)	100
Age category (*X*^2^: 71.47***)					
18–30	328	(19.33)	249.2	(18.5)	20.04
31–40	324	(19.09)	245.8	(18.25)	17.05
41–50	266	(15.67)	201.6	(14.97)	15.79
51–60	262	(15.44)	214.2	(15.9)	15.84
61–70	256	(15.09)	217.6	(16.15)	13.3
71–80	235	(13.85)	197.6	(14.67)	11.97
81–90	25	(1.47)	20	(1.48)	5.06
91–100	1	(0.06)	1	(0.07)	0.96
Total	1697	(100)	1347	(100)	100
Survey phase					
Stage 2	199	(11.73)	155.4	(11.54)	
Stage 3	1498	(88.27)	1192	(88.46)	
Total	1697	(100)	1347	(100)	

N-weighted is the effective sample size of the TTO data after excluding 20% of participants with the poorest CIS scores and 53 TTO answers due to technical issues. The weighting corresponds to the included numbers of TTO answers per participant (100% = 5 TTO answers). The last column shows the population distribution in proportions from Statistics Sweden if available

The title row of each categorical category, where comparable population data is available, reports the results of a Chi-squared test for difference against population proportions. Stars signify significance levels: * for 0.05, ** for 0.01, and *** for 0.001. If no degree of freedom (*df*) is reported, simulated p values were used

Time trade-off (TTO). Combined inconsistency severity (CIS)

A total of 17% of the participants failed the DCE consistency check (288 out of 1697). In the TTO questions, 50% (61.7% after exclusions) of all participants had no strict inconsistent answer, 23.7% (27.3%) had two answers involved in strict inconsistencies, and 26.3% (11%) had three or more answers involved in strict inconsistencies (Supplementary Table S6). The frequent valuations of higher capability states at 0.1, and relatively low TTO mean answers of 0.55 at LSS 15 values (Fig. 2) may indicate limited data quality.

### Modeling

**Fig. 3:**
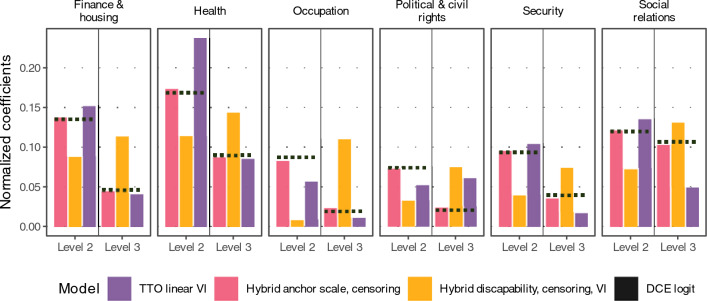
Comparison of coefficient magnitude per model. Bars represent the coefficient magnitude for level attributes, in comparison to the DCE logit coefficients (Dotted horizontal lines). Normalized per model, so that the sum of the coefficient without the intercepts corresponds to 100%. Varying intercept (VI). *DCE* discrete choice experiment

The summed coefficients per attribute (Table 2) indicated that the attribute importance order was largely stable across the models with health valued highest, followed by social relations, finance and housing, security, political and civil rights, and occupation. For the logit model and the TTO models with a constant, for all attributes except social relations, the step from level 1 to level 2 was substantially higher than the step from level 2 to level 3. For social relations, the logit model and the hybrid model with attainment coding indicated that both steps from level 1 to 2 and from level 2 to 3 were rather equally important, while the TTO linear model also showed a reduced gain from level 2 to 3 (Table 2, Fig. 3).

**Table 2:** Model comparison

	TTO linear VI	Hybrid	Hybrid anchor scale	Hybrid anchor scale, censoring
Don’t agree (constant)	0.214 (0.2, 0.23)	0.215 (0.2, 0.23)	0.216 (0.21, 0.23)	0.114 (0.1, 0.13)
Health	Rank 1	Rank 1	Rank 1	Rank 1
Agree partially	0.09 (0.08, 0.1)	0.068 (0.06, 0.07)	0.137 (0.13, 0.15)	0.154 (0.14, 0.17)
Agree completely	0.032 (0.02, 0.04)	0.035 (0.03, 0.04)	0.07 (0.06, 0.08)	0.078 (0.07, 0.09)
Social relations	Rank 3	Rank 2	Rank 2	Rank 2
Agree partially	0.051 (0.04, 0.06)	0.048 (0.04, 0.05)	0.096 (0.09, 0.11)	0.108 (0.1, 0.12)
Agree completely	0.019 (0.01, 0.03)	0.04 (0.04, 0.04)	0.08 (0.07, 0.09)	0.091 (0.08, 0.1)
Finance & housing	Rank 2	Rank 3	Rank 3	Rank 3
Agree partially	0.058 (0.05, 0.07)	0.054 (0.05, 0.06)	0.108 (0.1, 0.12)	0.122 (0.11, 0.13)
Agree completely	0.016 (0, 0.03)	0.017 (0.01, 0.02)	0.035 (0.03, 0.04)	0.04 (0.03, 0.05)
Occupation	Rank 6	Rank 6	Rank 6	Rank 6
Agree partially	0.022 (0.01, 0.03)	0.033 (0.03, 0.04)	0.066 (0.06, 0.08)	0.073 (0.06, 0.08)
Agree completely	0.004 (0, 0.01)	0.008 (0, 0.01)	0.016 (0.01, 0.03)	0.021 (0.01, 0.03)
Security	Rank 4	Rank 4	Rank 4	Rank 4
Agree partially	0.039 (0.03, 0.05)	0.037 (0.03, 0.04)	0.075 (0.06, 0.08)	0.085 (0.07, 0.1)
Agree completely	0.007 (0, 0.02)	0.014 (0.01, 0.02)	0.028 (0.02, 0.04)	0.031 (0.02, 0.04)
Political & civil rights	Rank 5	Rank 5	Rank 5	Rank 5
Agree partially	0.02 (0.01, 0.03)	0.028 (0.02, 0.03)	0.056 (0.05, 0.07)	0.065 (0.05, 0.08)
Agree completely	0.023 (0.01, 0.03)	0.01 (0.01, 0.02)	0.021 (0.01, 0.03)	0.021 (0.01, 0.03)
Linear model SD	0.176 (0.17, 0.18)	0.237 (0.23, 0.24)	0.237 (0.23, 0.24)	0.302 (0.3, 0.31)
Varying intercept SD	0.158 (0.15, 0.17)			
Logit scale factor		13.418 (12.5, 14.45)	6.703 (6.3, 7.1)	5.952 (5.6, 6.32)
Anchor scale			0.497 (0.47, 0.52)	0.545 (0.52, 0.57)
K-fold				
MAE	0.196	0.196	0.196	0.195
MSE	0.056	0.056	0.056	0.06
Accuracy	72.198	73.277	73.345	73.345
Other model properties				
N observations	6735	27,099	27,099	27,099
N observations DCE	0	20,364	20,364	20,364
N observations TTO	6735	6735	6735	6735
Range	0.381	0.393	0.785	0.887
333333	0.596	0.608	1.001	1.001
111111	0.215	0.215	0.216	0.114

Model coefficients and properties for selected models

MAE and MSE are reported on the original answer scale (with applied multiplicative anchor scale) for the two models in the right-hand side columns, for comparability with the unscaled coefficients of the models in the two left-hand side columns. The rank refers to the magnitude of the added coefficients per attribute

*SD* standard deviation, *MSE* mean squared error, *MAE* mean absolute error, *CI* credible interval

**Fig. 4:**
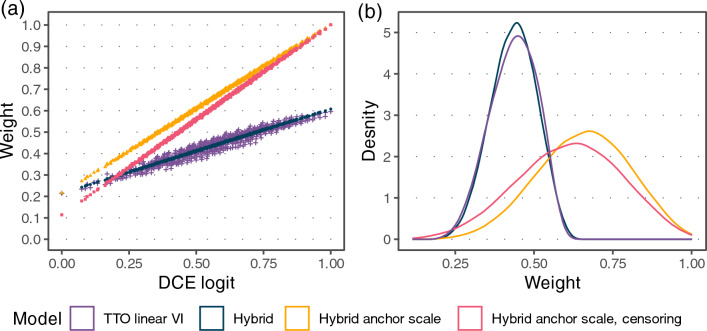
Weight distributions. **a** Scatter plot of weights for, depicting the agreement of weights generated by different models with the normalized DCE logit weights. **b** Density of weights on the [0, 1] interval for different models. TTO time trade-off, DCE discrete choice experiment

Stretched coefficients for level 3 and compressed coefficients for level 2 occurred for the discapability specification without a constant compared to the DCE logit model. The stretching stems from the higher density of TTO answers towards the lower end of the [0, 1] interval (Fig. 1). As a result, the order of the level 2 and 3 coefficients compared to the DCE logit model was effectively reversed (Fig. 3). In comparison, the hybrid model coefficients largely corresponded to the DCE logit coefficients. This was also reflected in the resulting distribution of weights where the hybrid models in the attainment specification correlated more with the DCE logit weights than TTO-only models or models in the discapability specification (Fig. 4).

### Model comparison

The hybrid models in the attainment specification with anchor scales and with censoring scale were ranked higher in the average percentile ranking than discapability specification models and TTO data-only models. Lower ranks of models in discapability coding and models with only TTO data were driven by lower DCE accuracy, lower DCE logit fidelity, and partially lower precision in terms of mean credible interval widths for coefficients and weights. We chose to generate the final value set with the model with the anchor scale and censoring. The models using the heteroskedasticity specification or varying intercept did not show improved performance and were less parsimonious, while censoring improved the range (Supplementary Table S7, Fig. S8, S9). The resulting weights of the final model stretched from 0.114 to 1.001 (Table 2, Fig. 4, Supplementary Table S9).

The sensitivity analysis showed that, compared to including 80%, including 90% or 100% of the TTO data resulted in lower coefficients and slightly higher mean 95% CI widths and higher kfold MAE relative to the range. Relative to the range, including 50% to 70% of the TTO data showed slightly improved MAE, but only a minor decrease in terms of the mean 95% CI widths of the weights and coefficients (Supplementary Table S8).

## Discussion

We produced a value set for CALY-SWE using an online survey among a partially representative general population sample of Swedish participants and a TTO and DCE data hybrid model to generate the weights. This value set enables CALY-SWE to be used in economic evaluations.

This is the first study eliciting a value set for the CALY-SWE instrument and the first application of the DCE and TTO hybrid modeling method for a QoL instrument outside the health sphere.

### Comparison to other studies

In a Swedish study using an earlier version of the capability list (with financial situation and housing as separate attributes), that informed the CALY-SWE Delphi process, health, social relations, and financial situation were ranked highest [68]; thus corroborating the present ranking. ICECAP-A is another general population capability instruments where a tariff is available. While a complete comparison is challenging due to the differing attributes [66], the ‘attachment’ dimension, which may be comparable to social relations, showed the largest coefficients. Here, social relations were also valued second most important after health (which is not an independent attribute in ICECAP-A).

Despite the methodological similarity with EQ-5D-5L weight valuations, there are also key differences. The capability approach is reflected in the attainment phrasing of the statements as opposed to dis-utility in health. Together with the different TTO iteration procedure, these are likely to be contributing factors to the clustering of answers in the middle and bottom of the [0, 1] interval (Fig. 1) compared to EQ-5D-5L valuations where TTO answers also cluster close to 1. Meanwhile, our TTO data showed comparingly less extreme clustering [34, 35, 38, 60, 69, 70]. Consequentially, the model coefficients result in a weight for 333333 below 1, which is at odds with the TTO task that implies a weight of 1. While the inclusion of the anchor scale rectified this issue, the resulting weights might not truly reflect the TTO data. Constraining a model without a constant to the data as an alternative would neither solve this issue nor necessarily result in the same preference ordering as the naive TTO or DCE logit model. Importantly, the anchor scale does not alter the order of levels and attributes and leaves the pit state anchoring unchanged, which is preferable given the relevance for resource allocation decisions.

### Strengths and limitations

Strengths include a careful development of the survey to improve data quality, and representative sampling according to region and gender. Furthermore, the exclusion of low-quality TTO data based on a sensitive exclusion criterion allowed the value set to be estimated with greater precision which was corroborated by a sensitivity analysis. We also integrated complementary DCE and TTO preference data. The value set generated by a hybrid model maintains the preference order of attributes and levels revealed by the arguably less-biased DCE data, and we included the anchor scale factor in the model to map the weight of 333333 to 1 to maintain the value set’s face validity.

Limitations of this evaluation include indications of TTO data quality issues despite efforts to adopt the survey to fit the unsupervised online mode. Online sampling through a panel does not guarantee the same level of data transparency and trustworthiness as person-to-person interviews. Yet, unobserved participation may reduce social desirability bias and enable participants to state genuine preferences. The sample’s representativeness was limited, with bias towards higher education, younger age, and Swedish born participants compared to the population. The exclusion of data may have further reduced representativeness, although we did not find evidence for substantial differences to the overall sample. The remaining effective TTO sample size of 1347 participants exceeded the targeted minimal sample size of 500 for the hybrid model.

Excluding data can also be morally justified because the weight valuation study constitutes a democratic process for measuring normative values for legitimately guiding societal resource allocation. Priority setting should be laid in the hands of “fair-minded people” [71]. Assuming rationality, data from participants that answer the TTO questions sincerely should be considered before participants with more inconsistent answers. Even non-directional bias connected to lower data quality could otherwise affect precision and attribute level preferences. For DCE, we emphasized data quality to a lesser extent, however a proportion of 17% inconsistent answers is comparable to other studies [72]. Potentially, an order bias occurred because the DCE block preceded the TTO block, but the nature and direction of bias is difficult to assess because of the differences in the DCE and TTO format.

Further, because we only examined main effects, we may have missed plausible interactions, for example, for health and social relations or for occupation and financial situation and housing. However, we focused on producing an initial CALY-SWE value set that can be widely used and easily interpreted.

Another limitation concerns the TTO answer range. To limit the length of the survey and the cognitive burden for participants, we restricted answers to integers between 1 and 10 years, possibly reducing the precision. This range did not allow for valuations below 0 either, contrary to many QALY valuation studies [5, 59], and 0 was anchored to 0 lifetime. Worse-than-death TTO valuations come with methodological difficulties and increased complexity [73]. Relatedly, anchoring death to 0 may be ethically controversial because death may not be a morally acceptable comparator for very low capability. For health, in comparison, death may be naturally related to severe ill-health [12]. An alternative approach, adopted for ICECAP [66], is to anchor 0 to the pit state, but this arguably suffers from similar ethical issues where living in 111111 implies no capability-adjusted lifetime. Future research needs to investigate and clarify the pit-state state anchoring in a capability context, in conjunction with improving TTO tasks in online surveys.

## Conclusion

We elicited a CALY-SWE value set that can be used to measure CALYs in economic evaluations of interventions with social consequences beyond health. Health, social relations, and finance and housing where valued highest, followed by occupation, security, and political and civil rights. The tariff is indicative of the Swedish general population’s preferences, and the facilitated measurement of capabilities may be relevant for policy decisions with societal consequences.

### Supplementary Information

Below is the link to the electronic supplementary material.

Supplementary file1 (DOCX 4670 KB)

## Data Availability

The data supporting the findings of the study are available upon reasonable request via the Swedish National Data Service (SND) at 10.5878/asxy-3p37.
