# Supplementary material for: Eliciting a value set for the Swedish Capability-Adjusted Life Years instrument (CALY-SWE)
Source: Qual Life Res. 2023 Sep 11;33(1):59–72. doi: 10.1007/s11136-023-03507-w (PMC10784385; doi:10.1007/s11136-023-03507-w)
Supplement: Supplementary file 1 — Supplementary file1 (DOCX 4670 KB) [file 11136_2023_3507_MOESM1_ESM.docx]

Contents

[CALY-SWE Instrument 3](#_Toc140848081)

[Survey Screenshots 5](#_Toc140848082)

[Iteration procedure 8](#_Toc140848083)

[Income distribution 9](#_Toc140848084)

[Background characteristics excluded participants 10](#_Toc140848085)

[Timing 14](#_Toc140848086)

[TTO answers per state and level sum score 15](#_Toc140848087)

[Inconsistencies 25](#_Toc140848088)

[Definition 25](#_Toc140848089)

[Distribution 25](#_Toc140848090)

[Combined inconsistency severity (CIS) score 25](#_Toc140848091)

[Calculation 25](#_Toc140848092)

[Distribution CIS score 27](#_Toc140848093)

[Model specifications 27](#_Toc140848094)

[Stan code final model 28](#_Toc140848095)

[Extended model results 30](#_Toc140848096)

[Predicted vs observed weights 32](#_Toc140848097)

[Results sensitivity analyses 34](#_Toc140848098)

[Weight table final model 38](#_Toc140848099)

*Supplementary Information for article*: Eliciting a value set for the Swedish Capability-Adjusted Life Years instrument (CALY-SWE)

*Author names*: Kaspar Walter Meili, Brendan Mulhern, Richard Ssegonja, Fredrik Norström, Inna Feldman, Anna Månsdotter, Jan Hjelte, Lars Lindholm

*Corresponding author*: Kaspar Walter Meili, Department of Epidemiology and Global Health, Umeå University

# CALY-SWE Instrument

| **What** | **English (informal translation)** | **Swedish** |
| --- | --- | --- |
| - Hälsa - Health | I have in general good health (mental and physical) that almost always (more than 95% of days) allows me to work or do what I want. | Jag har ett bra allmänt hälsotillstånd (psykiskt och fysiskt) som nästan alltid (säg minst 95% av alla dagar) tillåter mig att arbeta eller ägna mig åt det jag vill. |
| - Nära relationer - Social relations | I have access to close relations (family, friends, or acquaintances) who give me advice and support when I need. | Jag har tillgång till nära relationer (familj, vänner eller bekanta) som ger mig råd och stöd när jag behöver. |
| - Ekonomi och boende - Finance and housing | I have a financial situation (salary, other incomes, savings) that always allows me to have a permanent housing, and mostly (at least 8 out of 10 times) buy what I think I need. | Jag har en ekonomi (lön, annan inkomst eller besparingar) som alltid tillåter mig att ha en fast bostad och för det mesta (minst 8 gånger av 10) tillåter mig att köpa det jag tycker mig behöva. |
| - Sysselsättning - Occupation | I have an employment or another occupation (study, internship, homework, care of relative) that I am mostly satisfied with. During the last five years I have been satisfied with my occupation at least 75% of the time. | Jag har ett arbete eller annan sysselsättning (studier, praktik, hemarbete, vård av anhörig, etc.) som jag för det mesta är nöjd med. De senaste fem åren har jag minst 75% av tiden varit nöjd med det jag ägnat mig åt. |
| - Säkerhet - Security | I have a sense of security, and the risk of violence or other crime does not influence me in my work or leisure activities. | Jag har en trygghet som gör att risk för våld eller annan kriminalitet inte påverkar mig i mitt arbete eller i min fritid. |
| - Politiska och medborgerliga rättligheter - Political and civil rights | I trust that our rights as citizens, and our active participation in the democratic processes make it possible to shape our common living conditions. | Jag litar på att våra rättigheter som medborgare, och vårt aktiva deltagande i det demokratiska systemet, gör det möjligt att påverka våra gemensamma livsvillkor |
| Level 1 answer | Do not agree | Instämmer inte |
| Level 2 answer | Partially agree | Instämmer delvis |
| Level 3 answer | Completely agree | Instämmer helt |

Table S1. CALY-SWE instrument. Phrasing and answer alternative labels. Swedish and informal English translations.

# Survey Screenshots

Fig S1. Screenshots CALY-SWE statements and questions. Swedish left, English right (informal translation).


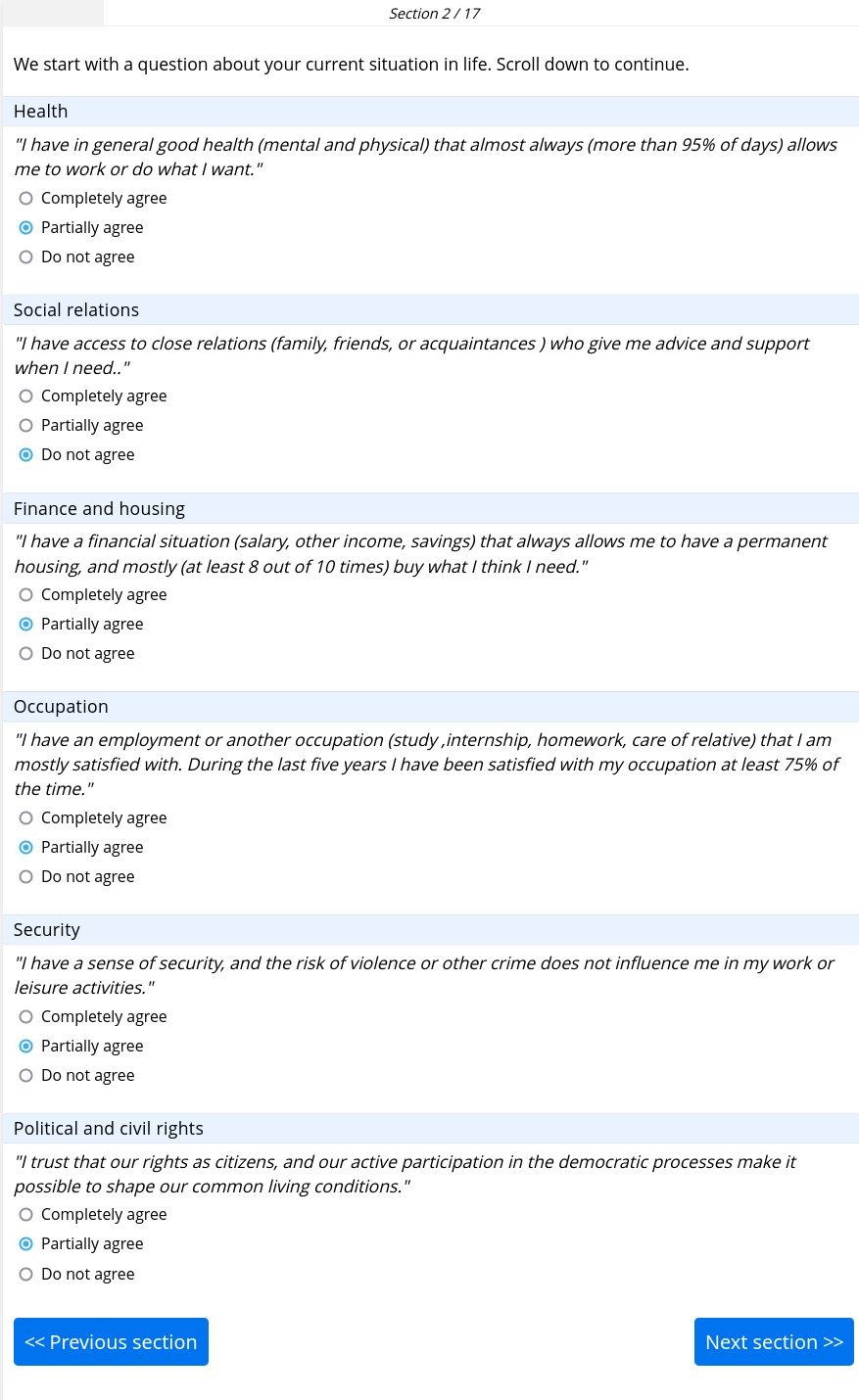

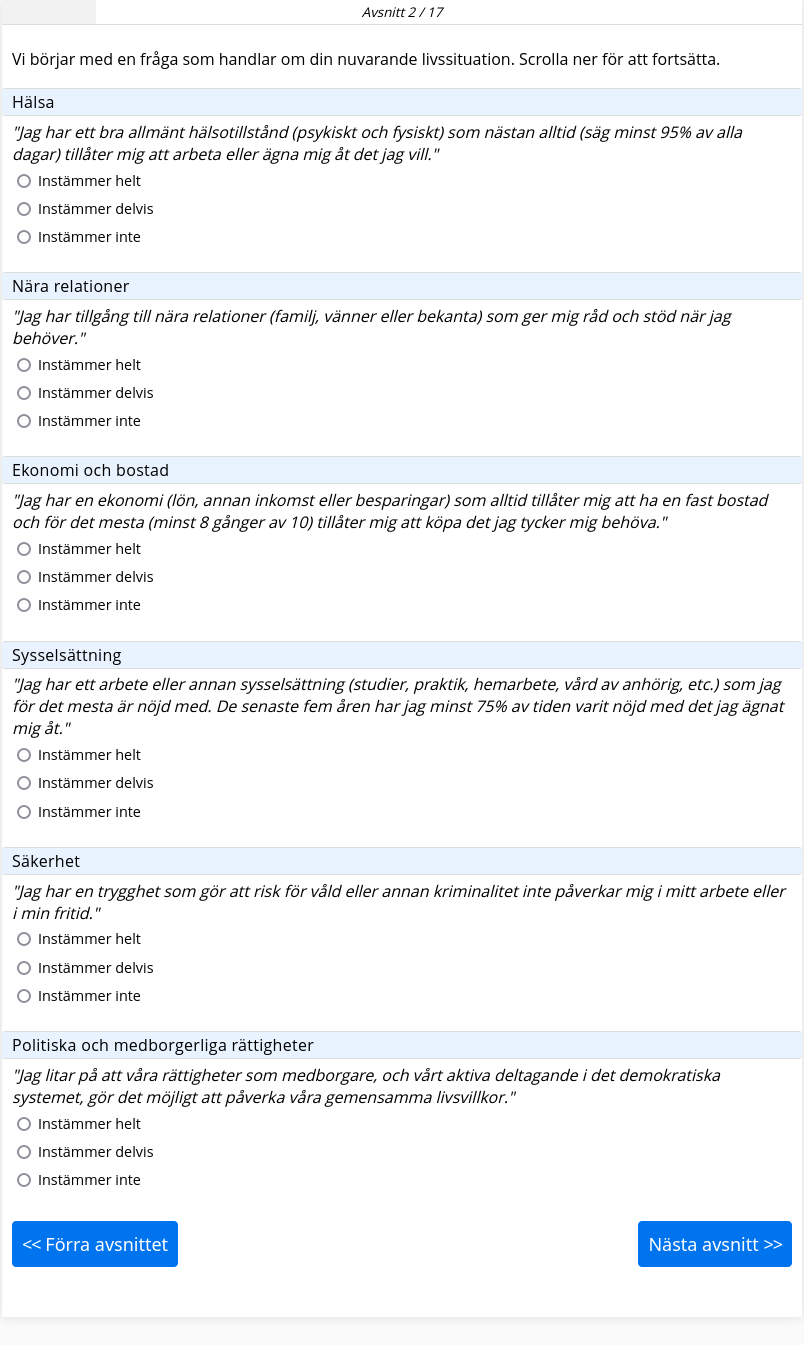


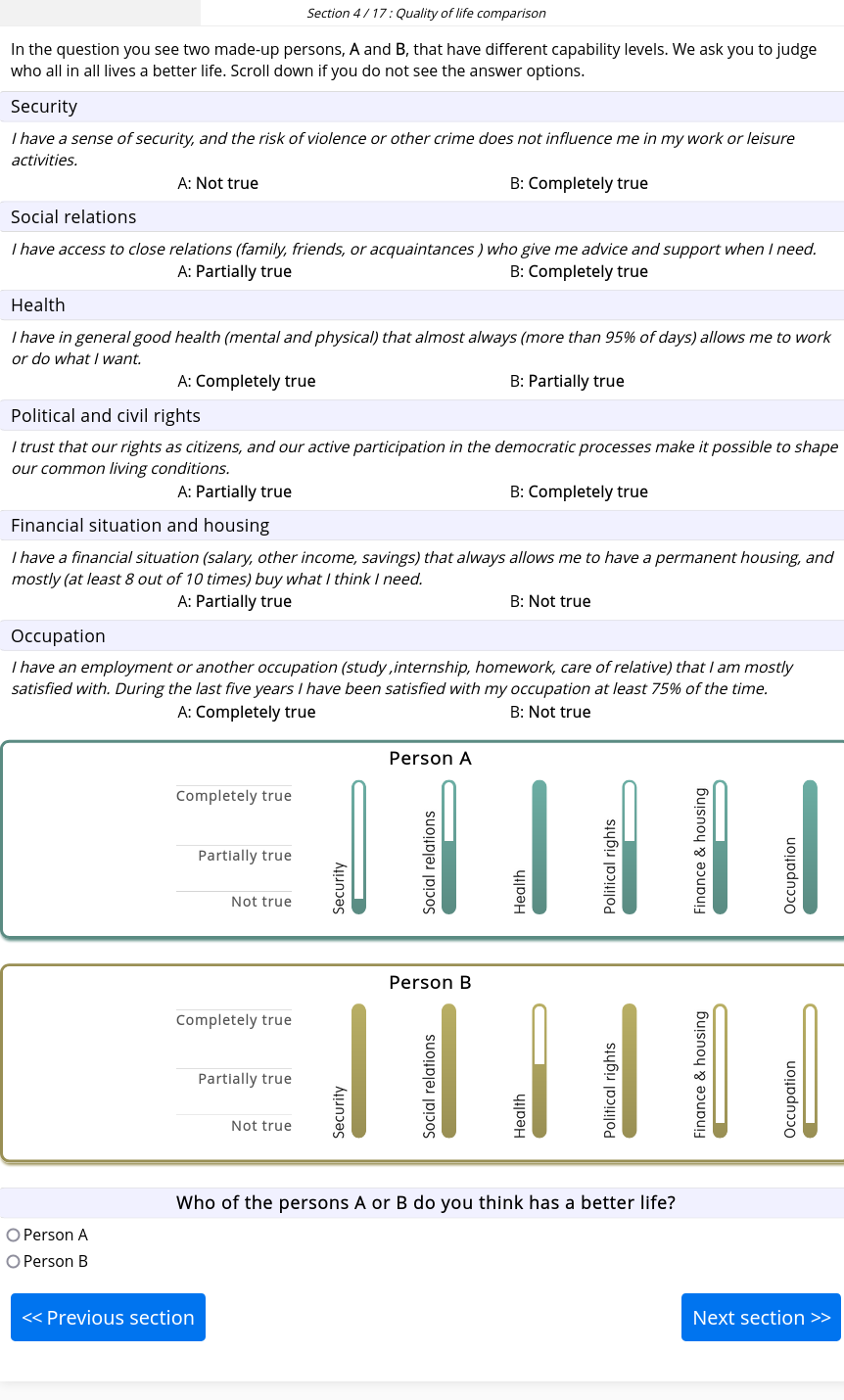

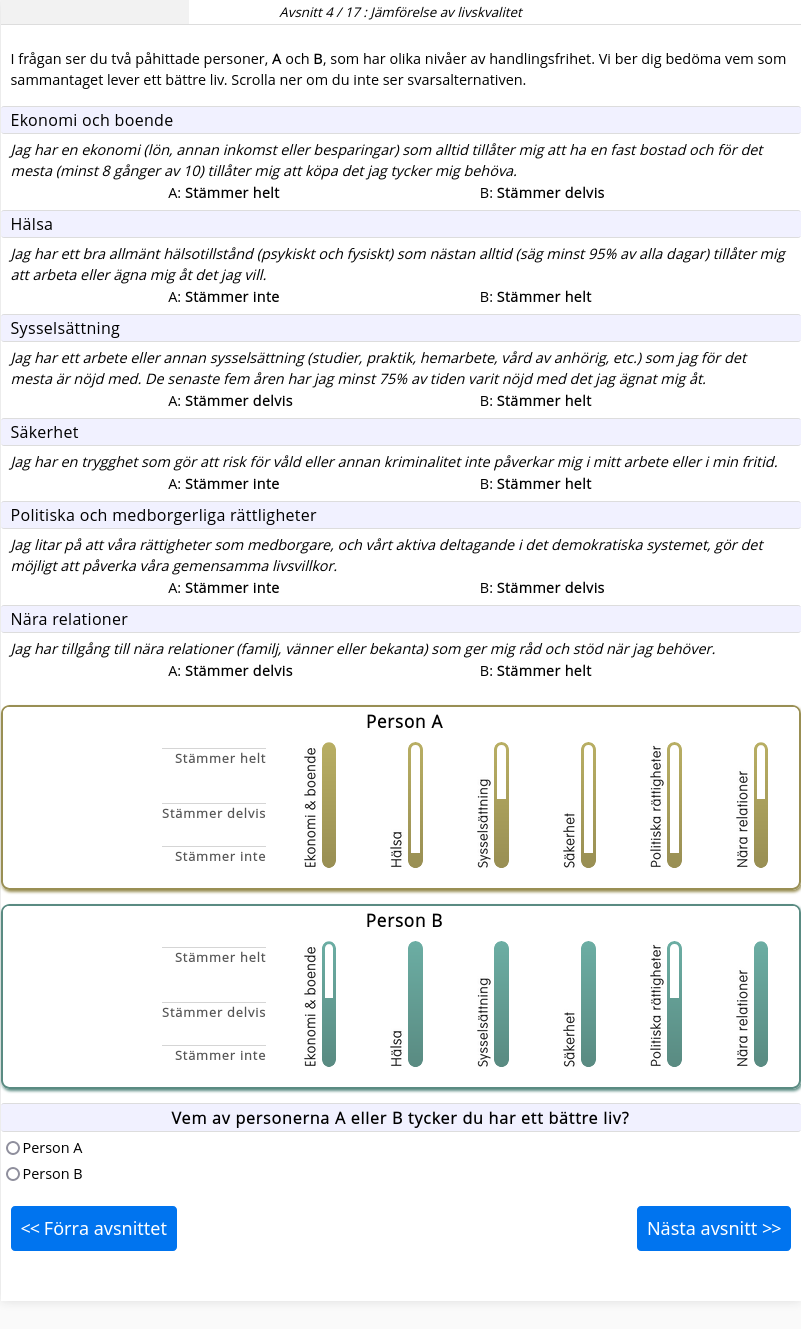


Fig S2. Screenshots DCE question. Swedish left. English right (informal translation).

Fig S3. Screenshots TTO question. Left: First learning state iteration. Middle: Second learning state iteration. Right: Normal TTO iteration. Swedish on top. English on the bottom (informal translation).


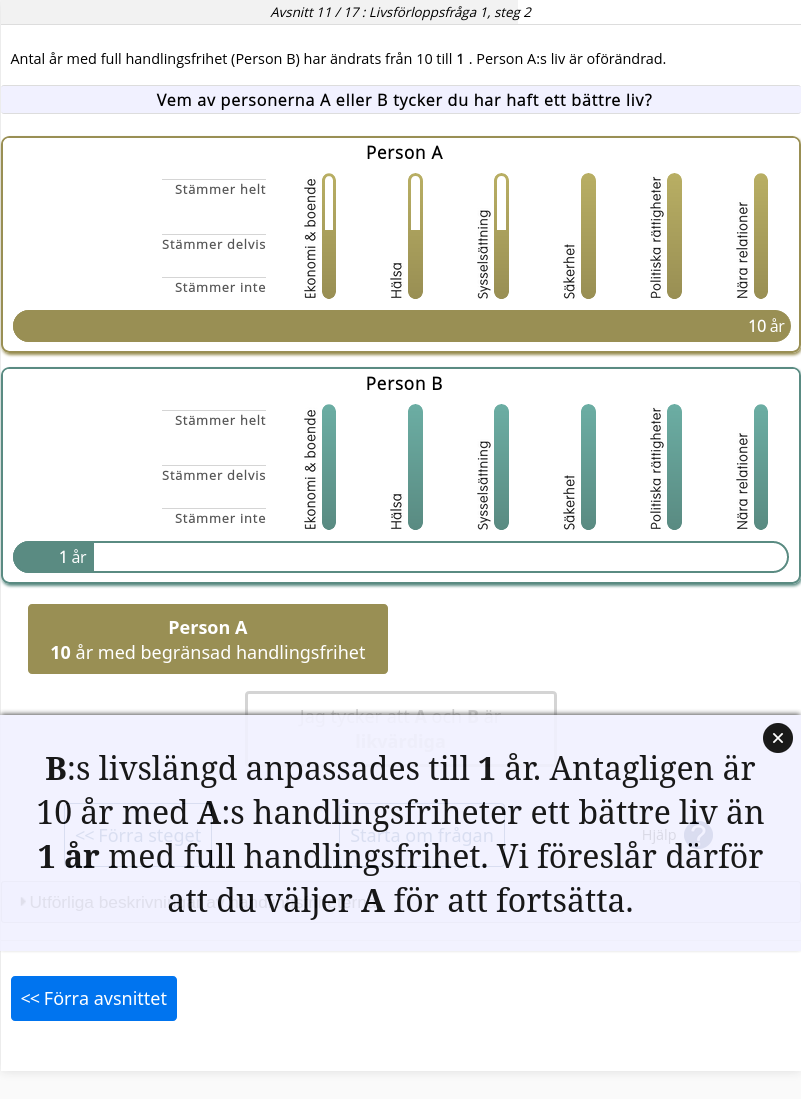

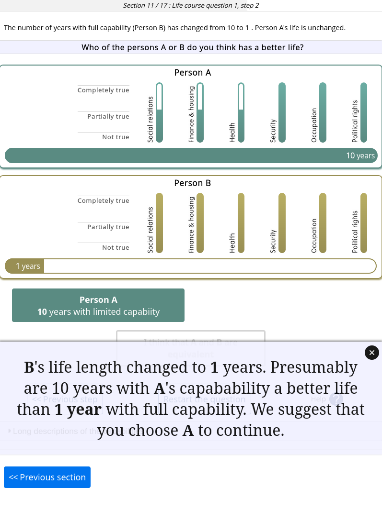

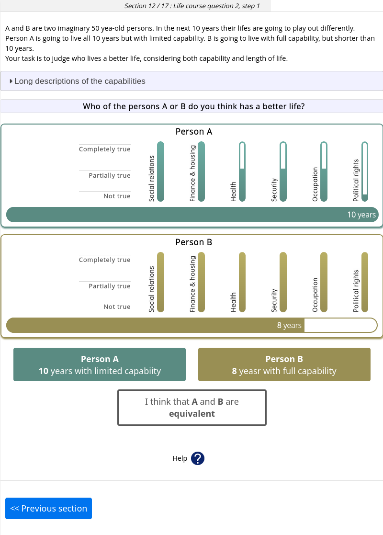

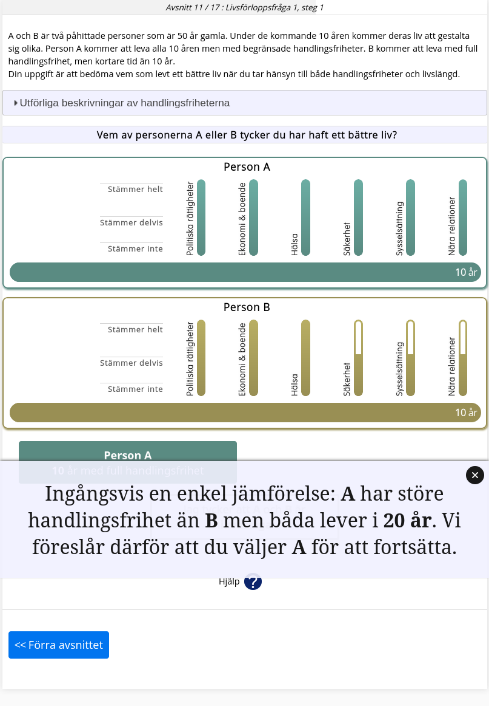

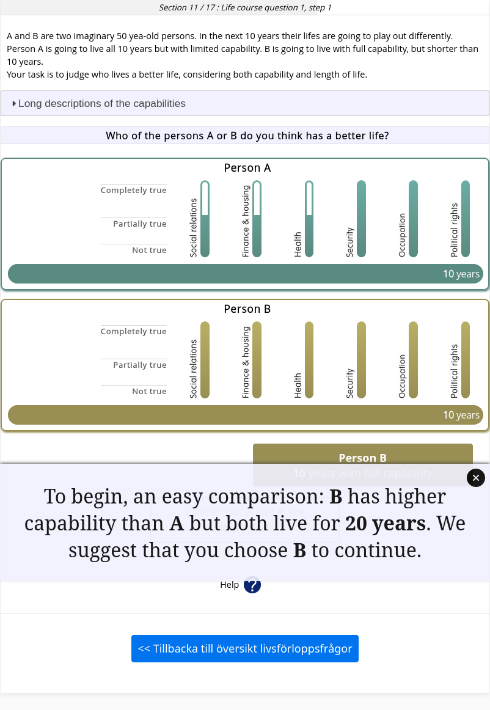

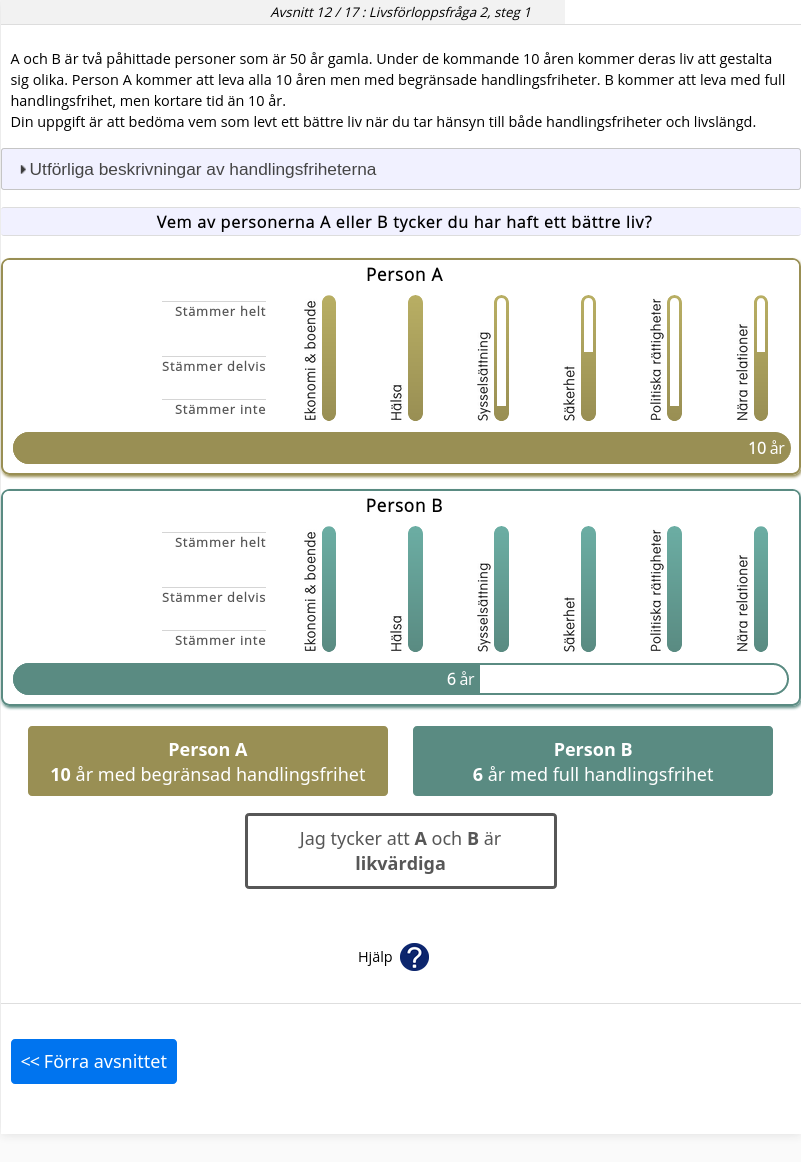


# Iteration procedure

To avoid clustering, we adopted a random bisection iteration procedure with a random starting point as the initial bisection value (which we refer to as ‘shedvalue’):

- If participants preferred the *limited capability* state (with the full time span of 10 years), a new random shedvalue was chosen between the current shedvalue value +1 and the current upper interval limit (starting limit of 10 years), making the limited capability state less attractive and the full capability state more attractive
- If participants preferred the *full capability* state (with tradeable duration), a new random shedvalue was chosen between the current shedvalue value -1 and the current lower interval limit (starting limit 1), making the limited capability state more attractive and the full capability state less attractive
- For the above two alternatives (full capability, limited capability), for choosing a new shedvalue, values at the limits of the current interval were avoided if possible (avoidance of a margin of 20% of the interval, or 1, whatever was higher). If the new interval had width 1 (2 possible values), the shedvalue was chosen to not be identical to the shedvalue of the last iteration.
- Participants could also choose *equal*, the new bisection interval was then narrowed to half of the width of the current bisection interval: The upper interval limit was set at the current shedvalue+ ¼ * current bisection interval with, and the lower limit to the current shedvalue – ¼ * the current bisection interval width. If the new limits were the same or went beyond the old limits on either side, they were set so that the interval became one integer narrower on the respective side.
- If just one possible value was left for the new interval (bisection interval width 0), participants were prompted with a confirmation question to confirm their choice of equivalence.

Rounding-up for widths and margins was used in case of fractional values.

The first two iterations for the learning states were guided, giving the participants only one option (first a comparison between 10 years in full capability and 10 years in slightly limited capability, then a comparison between 1 year in full capability and 10 years in slightly limited capability, see survey screenshots). After the guided iterations, the above-described iteration procedure was used with a starting bisection interval from 2 to 9 years.

Example:

*Iteration x: (lower limit, shedvalue, upper limit)*

Iteration 1: (1,6,10) -> Participant indicates *equal*

Iteration 2: (3,3,9) -> Participant prefers *limited capability*

Iteration 3: (4,7,9) -> Participant prefers *full capability*

Iteration 4: (4,5,6) -> Participant indicates *equal*

Iteration 5: (5,5,5) -> Confirmation question displayed

# Income distribution

Because the income brackets used by Statistics Sweden and in the survey differed, it was not possible to directly compare the number of people in each bracket. Here we thus present the income mass distribution for SCB and the survey sample with the respective set of income categories.


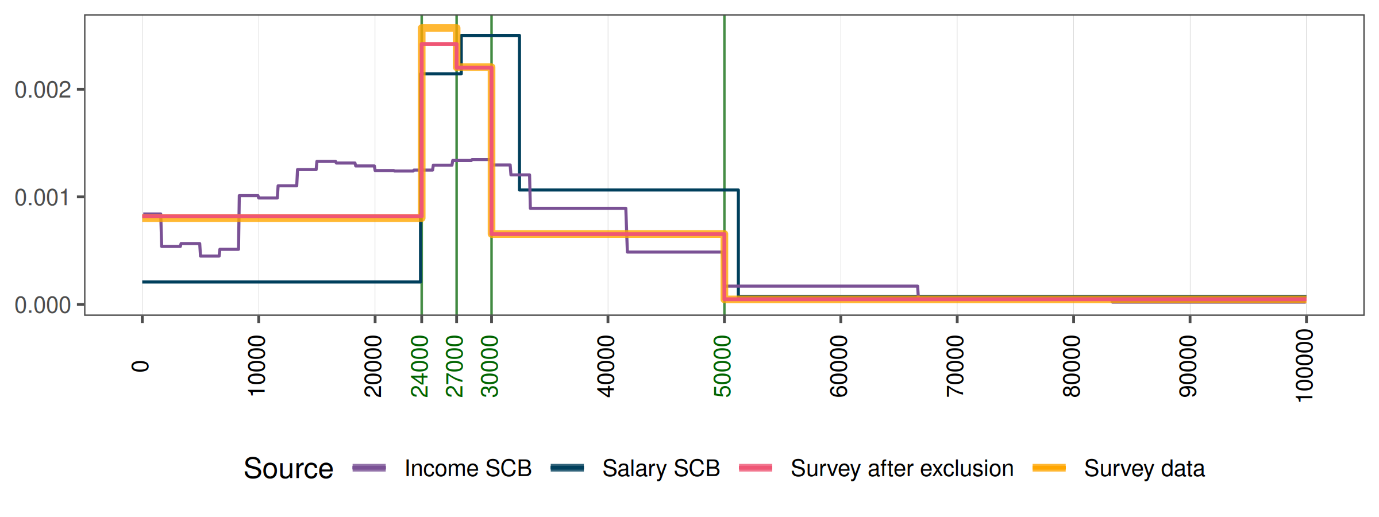


Fig S4. Probability mass income distribution brackets. Monthly income in Swedish Kronor is displayed on the x axis, probability density on y axis. Salary SCB and Income SCB display the salary distribution and income distribution obtained from Statics Sweden. *After exclusion* refers to the income distribution after excluding TTO data from the 20% of participants with worst inconsistency scores and 53 TTO answers due to technical issues. Time trade-off (TTO).

# Background characteristics excluded participants

| **Category** | **N sample** | **P sample %** | **N Excluded TTO** | **P Excluded TTO%** | **Percentage point diff. excluded vs sample** |
| --- | --- | --- | --- | --- | --- |
|  |  |  |  |  |  |
| *Age* |  |  |  |  |  |
| Mean (1st quartile, 3rd quartile) | 48.73 (33, 64) |  | 45.79 (33, 64) |  |  |
|  |  |  |  |  |  |
| *Birthplace (X²: 97.5)* |  |  |  |  |  |
| Sweden | 1529 | 90.47% | 287 | 85.32% | 5.15 |
| Other Nordic country | 33 | 1.95% | 7 | 2.08% | -0.13 |
| Europe (West) | 21 | 1.24% | 4.8 | 1.43% | -0.19 |
| Europe (South) | 16 | 0.95% | 3 | 0.89% | 0.06 |
| Europe (East) | 27 | 1.6% | 8 | 2.38% | -0.78 |
| Africa | 8 | 0.47% | 5 | 1.49% | -1.02 |
| Western Asia | 28 | 1.66% | 12 | 3.57% | -1.91 |
| Southern Asia | 4 | 0.24% | 1 | 0.3% | -0.06 |
| Southeast Asia | 4 | 0.24% | 2.6 | 0.77% | -0.53 |
| Eastern Asia | 6 | 0.36% | 1 | 0.3% | 0.06 |
| North America | 4 | 0.24% | 1 | 0.3% | -0.06 |
| Latin America | 4 | 0.24% |  |  |  |
| Rest of the world | 6 | 0.36% | 4 | 1.19% | -0.83 |
| Abstain | 7 |  | 3 |  |  |
| Total | 1697 | 100% | 339.4 | 100% | 0 |
|  |  |  |  |  |  |
| *Region (X²: 56)* |  |  |  |  |  |
| Stockholm | 357 | 21.05% | 69.6 | 20.69% | 0.36 |
| East-Central Sweden | 286 | 16.86% | 64.8 | 19.26% | -2.4 |
| Småland and islands | 150 | 8.84% | 30 | 8.92% | -0.08 |
| South Sweden | 251 | 14.8% | 50 | 14.86% | -0.06 |
| West Sweden | 322 | 18.99% | 55 | 16.35% | 2.64 |
| North-Central Sweden | 155 | 9.14% | 33 | 9.81% | -0.67 |
| Central Norrland | 69 | 4.07% | 18 | 5.35% | -1.28 |
| Upper Norrland | 99 | 5.84% | 16 | 4.76% | 1.08 |
| NA | 7 | 0.41% | 2 |  |  |
| Total | 1696 | 100% | 338.4 | 100% | 0 |
|  |  |  |  |  |  |
| *Education (X²: 30)* |  |  |  |  |  |
| Less than 9 years schooling | 31 | 1.83% | 8 | 2.37% | -0.54 |
| Finished elementary (9 years) | 132 | 7.8% | 34 | 10.08% | -2.28 |
| High school or vocational (2 years) | 225 | 13.3% | 37 | 10.97% | 2.33 |
| High school (3-4 years) | 463 | 27.36% | 86.6 | 25.67% | 1.69 |
| Tertiary education (shorter than 3 years) | 346 | 20.45% | 80.8 | 23.95% | -3.5 |
| Tertiary education (3 years or more) | 495 | 29.26% | 91 | 26.97% | 2.29 |
| Abstain | 5 |  | 2 |  |  |
| Total | 1697 | 100% | 339.4 | 100% | 0 |
|  |  |  |  |  |  |
| *Gender (X²: 2)* |  |  |  |  |  |
| Woman | 846 | 50.36% | 166.6 | 49.82% | 0.54 |
| Man | 834 | 49.64% | 167.8 | 50.18% | -0.54 |
| Other | 9 |  | 1 |  |  |
| Abstain | 8 |  | 4 |  |  |
| Total | 1697 | 100% | 339.4 | 100% | 0 |
|  |  |  |  |  |  |
| *Housing (MC) (X²: 20)* |  |  |  |  |  |
| More or less without housing | 17 | 1.02% | 5 | 1.49% | -0.47 |
| Renting | 708 | 42.55% | 166.6 | 49.52% | -6.97 |
| Own an apartment | 330 | 19.83% | 59 | 17.54% | 2.29 |
| Own a house | 588 | 35.34% | 99.8 | 29.67% | 5.67 |
| Studentroom or shared living | 21 | 1.26% | 6 | 1.78% | -0.52 |
| Other | 47 |  | 4 |  |  |
| Abstain | 18 |  | 4 |  |  |
| Total | 1729 | 100% | 344.4 | 100% | 0 |
|  |  |  |  |  |  |
| *Income (X²: 20)* |  |  |  |  |  |
| Less than 24 000 SEK | 600 | 38.54% | 106.6 | 34.77% | 3.77 |
| 24 000 to 27 000 SEK | 240 | 15.41% | 59 | 19.24% | -3.83 |
| 27 000 to 30 000 SEK | 206 | 13.23% | 41 | 13.37% | -0.14 |
| 30 000 to 50 000 SEK | 408 | 26.2% | 82 | 26.74% | -0.54 |
| More than 50 000 SEK | 103 | 6.62% | 18 | 5.87% | 0.75 |
| Abstain | 140 |  | 32.8 |  |  |
| Total | 1697 | 100% | 339.4 | 100% | 0 |
|  |  |  |  |  |  |
| *Living situation (MC) (X²: 20)* |  |  |  |  |  |
| Single | 516 | 25.23% | 102.8 | 25.3% | -0.07 |
| With partner | 951 | 46.5% | 196.8 | 48.43% | -1.93 |
| With parents | 97 | 4.74% | 16.8 | 4.13% | 0.61 |
| With sibling | 36 | 1.76% | 4 | 0.98% | 0.78 |
| With children (own or other) | 445 | 21.76% | 86 | 21.16% | 0.6 |
| Other | 28 |  | 7 |  |  |
| Abstain | 7 |  | 1 |  |  |
| Total | 2080 | 100% | 414.4 | 100% | 0 |
|  |  |  |  |  |  |
| *Municipality size (X²: 20)* |  |  |  |  |  |
| Less than 20k inhabitants | 288 | 17.13% | 39 | 11.66% | 5.47 |
| 20k to 50k inhabitants | 295 | 17.55% | 60.8 | 18.18% | -0.63 |
| 50k to 100k inhabitants | 336 | 19.99% | 76 | 22.73% | -2.74 |
| 100k to 300k inhabitants | 338 | 20.11% | 56 | 16.75% | 3.36 |
| Big city (Stockholm, Gothenburg, Malmö) with > 300k inhabitants | 424 | 25.22% | 102.6 | 30.68% | -5.46 |
| Abstain | 16 |  | 5 |  |  |
| Total | 1697 | 100% | 339.4 | 100% | 0 |
|  |  |  |  |  |  |
| *Age category (X²: 48)* |  |  |  |  |  |
| 18-30 | 328 | 19.33% | 76.8 | 22.63% | -3.3 |
| 31-40 | 324 | 19.09% | 76.8 | 22.63% | -3.54 |
| 41-50 | 266 | 15.67% | 62.8 | 18.5% | -2.83 |
| 51-60 | 262 | 15.44% | 46 | 13.55% | 1.89 |
| 61-70 | 256 | 15.09% | 38 | 11.2% | 3.89 |
| 71-80 | 235 | 13.85% | 34 | 10.02% | 3.83 |
| 81-90 | 25 | 1.47% | 5 | 1.47% | 0 |
| 91-100 | 1 | 0.06% |  |  |  |
| Total | 1697 | 100% | 339.4 | 100% | 0 |
|  |  |  |  |  |  |
| *Age category Cint (X²: 42)* |  |  |  |  |  |
| 18-24 | 150 | 8.84% | 31.8 | 9.37% | -0.53 |
| 25-34 | 314 | 18.5% | 78 | 22.98% | -4.48 |
| 35-44 | 288 | 16.97% | 69.8 | 20.57% | -3.6 |
| 45-54 | 279 | 16.44% | 52.8 | 15.56% | 0.88 |
| 55-64 | 268 | 15.79% | 41 | 12.08% | 3.71 |
| 65-74 | 228 | 13.44% | 36 | 10.61% | 2.83 |
| 75-99 | 170 | 10.02% | 30 | 8.84% | 1.18 |
| Total | 1697 | 100% | 339.4 | 100% | 0 |
|  |  |  |  |  |  |
| *Survey phase (X²: 2)* |  |  |  |  |  |
| Pilot phase | 199 | 11.73% | 42 | 12.37% | -0.64 |
| Main phase | 1498 | 88.27% | 297.4 | 87.63% | 0.64 |
| Total | 1697 | 100% | 339.4 | 100% | 0 |

Table S2. Comparison included and excluded data.
*N sample*: All participants.
*N Excluded TTO*: Excluded participants with the worst inconsistency scores and 53 TTO answers due to technical issues. Weighted according to numbers of TTO answers per participant (100% = 5 TTO answers).
The *P* columns correspond to the respective proportions and *Percentage point diff. excluded vs sample* is the percentage point difference between the entire sample and excluded participants. *MC* denotes that multiple choices were possible in the respective category. The title row of each categorical category, where comparable population data is available reports, the results of a chi-squared test for difference against population proportions. Time trade-off (TTO), Multiple choice (MC).

# Timing

| **By question** | **Mean** | **Median** | **1st quartile** | **3rd quartile** |
| --- | --- | --- | --- | --- |
| Introduction | 14.65 | 5 | 3 | 12 |
| Selfrating | 58.03 | 46 | 29 | 67 |
| VAS | 13.83 | 11 | 8 | 16 |
| DCE 1 | 74.29 | 57 | 31 | 92 |
| DCE 2 | 32.71 | 23 | 13 | 38 |
| DCE 3 | 29.59 | 18 | 10 | 31 |
| DCE 4 | 23.69 | 16 | 9 | 27 |
| DCE 5 | 23.73 | 15 | 8 | 25 |
| DCE 6 | 23.27 | 14 | 8 | 24 |
| TTO Introduction | 62.88 | 38 | 12 | 84 |
| TTO 1 (Learning) | 128.75 | 96 | 63 | 144 |
| TTO 2 | 63.43 | 39 | 23 | 65 |
| TTO 3 | 49.72 | 30 | 19 | 49 |
| TTO 4 | 38.49 | 26 | 17 | 42 |
| TTO 5 | 48.22 | 24 | 16 | 38 |
| TTO Overview | 34.46 | 22 | 14 | 39 |
| Background | 43.50 | 37 | 27 | 51 |
|  |  |  |  |  |
| **By question type** |  |  |  |  |
| Informative text | 38.75 | 12 | 4 | 44 |
| Self-rating | 58.03 | 46 | 29 | 67 |
| VAS | 13.83 | 11 | 8 | 16 |
| DCE | 34.55 | 20 | 10 | 37 |
| TTO | 65.80 | 36 | 20 | 69 |
| TTO overview | 34.46 | 22 | 14 | 39 |
| Background | 43.50 | 37 | 27 | 51 |
|  |  |  |  |  |
| **Total** | 44.88 | 25 | 13 | 50 |

Table S3. Answer time for one question. Mean, median 1^st^, and 3^rd^ quartile, in seconds, per question, question type, and overall.

# TTO answers per state and level sum score

| **All data** | | | | | | | |  |  |  |  |  |
| --- | --- | --- | --- | --- | --- | --- | --- | --- | --- | --- | --- | --- |
| **LSS** | **Mean** | **Stdev** | **Min** | **Max** | **10%** | **25%** | **50%** | **75%** | **90%** | **% lowest** | **Mean its** | **N evals** |
| 6 | 0.30 | 0.25 | 0.1 | 1 | 0.10 | 0.10 | 0.20 | 0.40 | 0.70 | 0.40 | 4.11 | 1684 |
| 7 | 0.34 | 0.26 | 0.1 | 1 | 0.10 | 0.10 | 0.20 | 0.50 | 0.80 | 0.34 | 4.02 | 210 |
| 9 | 0.37 | 0.28 | 0.1 | 1 | 0.10 | 0.10 | 0.30 | 0.50 | 0.90 | 0.31 | 4.02 | 422 |
| 10 | 0.39 | 0.27 | 0.1 | 1 | 0.10 | 0.20 | 0.30 | 0.60 | 0.80 | 0.24 | 4.06 | 421 |
| 11 | 0.41 | 0.27 | 0.1 | 1 | 0.10 | 0.20 | 0.40 | 0.60 | 0.80 | 0.23 | 3.92 | 422 |
| 12 | 0.44 | 0.27 | 0.1 | 1 | 0.10 | 0.20 | 0.40 | 0.60 | 0.80 | 0.20 | 3.94 | 841 |
| 13 | 0.46 | 0.28 | 0.1 | 1 | 0.10 | 0.20 | 0.40 | 0.70 | 0.90 | 0.19 | 4.00 | 1054 |
| 14 | 0.50 | 0.28 | 0.1 | 1 | 0.10 | 0.30 | 0.50 | 0.70 | 0.90 | 0.15 | 4.05 | 842 |
| 15 | 0.55 | 0.23 | 0.1 | 1 | 0.20 | 0.40 | 0.60 | 0.70 | 0.90 | 0.13 | 6.11 | 1694 |
| **Included TTO data only** | | | | | | | |  |  |  |  |  |
| **LSS** | **Mean** | **Stdev** | **Min** | **Max** | **10%** | **25%** | **50%** | **75%** | **90%** | **% lowest** | **Mean its** | **N evals** |
| 6 | 0.22 | 0.17 | 0.1 | 0.9 | 0.10 | 0.10 | 0.20 | 0.30 | 0.50 | 0.49 | 4.07 | 1344 |
| 7 | 0.28 | 0.21 | 0.1 | 0.9 | 0.10 | 0.10 | 0.20 | 0.40 | 0.60 | 0.40 | 4.07 | 168 |
| 9 | 0.34 | 0.26 | 0.1 | 1 | 0.10 | 0.10 | 0.30 | 0.50 | 0.80 | 0.34 | 3.93 | 350 |
| 10 | 0.36 | 0.24 | 0.1 | 1 | 0.10 | 0.10 | 0.30 | 0.50 | 0.70 | 0.26 | 3.99 | 330 |
| 11 | 0.39 | 0.26 | 0.1 | 1 | 0.10 | 0.20 | 0.30 | 0.60 | 0.80 | 0.24 | 3.96 | 343 |
| 12 | 0.43 | 0.27 | 0.1 | 1 | 0.10 | 0.20 | 0.40 | 0.60 | 0.80 | 0.21 | 3.97 | 664 |
| 13 | 0.44 | 0.27 | 0.1 | 1 | 0.10 | 0.20 | 0.40 | 0.60 | 0.80 | 0.20 | 3.99 | 855 |
| 14 | 0.50 | 0.27 | 0.1 | 1 | 0.10 | 0.30 | 0.50 | 0.70 | 0.90 | 0.14 | 4.06 | 684 |
| 15 | 0.56 | 0.22 | 0.1 | 1 | 0.20 | 0.40 | 0.60 | 0.70 | 0.90 | 0.11 | 6.08 | 1354 |

Table S4. Descriptive statistics for TTO answers per LLS. All TTO data on the top and TTO data after excluding participants according to the combined inconsistency score on the bottom. ‘% at lowest’ indicates the % of valuations at the lowest possible value (0.2 for learning states, 0.1 otherwise).
Mean iterations (Mean its). Number of evaluations (N evals). Level sum score (LSS). Time trade-off (TTO). Standard deviation (Stdev).

| **All data** | | | | | | | |  |  |  |  |  |  |
| --- | --- | --- | --- | --- | --- | --- | --- | --- | --- | --- | --- | --- | --- |
| **Config** | **LSS** | **Mean** | **Stdev** | **Min** | **Max** | **10%** | **25%** | **50%** | **75%** | **90%** | **% lowest** | **Mean its** | **N evals** |
| 111111 | 6 | 0.30 | 0.25 | 0.1 | 1 | 0.1 | 0.10 | 0.20 | 0.40 | 0.70 | 0.40 | 4.11 | 1684 |
| 111121 | 7 | 0.34 | 0.26 | 0.1 | 1 | 0.1 | 0.10 | 0.20 | 0.50 | 0.80 | 0.34 | 4.02 | 210 |
| 122211 | 9 | 0.37 | 0.28 | 0.1 | 1 | 0.1 | 0.10 | 0.30 | 0.50 | 0.81 | 0.35 | 4.02 | 210 |
| 211113 | 9 | 0.38 | 0.28 | 0.1 | 1 | 0.1 | 0.10 | 0.30 | 0.53 | 0.90 | 0.28 | 4.03 | 212 |
| 321112 | 10 | 0.43 | 0.28 | 0.1 | 1 | 0.1 | 0.20 | 0.40 | 0.60 | 0.80 | 0.22 | 4.07 | 211 |
| 113212 | 10 | 0.36 | 0.25 | 0.1 | 1 | 0.1 | 0.10 | 0.30 | 0.50 | 0.70 | 0.26 | 4.05 | 210 |
| 213311 | 11 | 0.39 | 0.27 | 0.1 | 1 | 0.1 | 0.10 | 0.30 | 0.60 | 0.80 | 0.26 | 3.85 | 212 |
| 212312 | 11 | 0.43 | 0.27 | 0.1 | 1 | 0.1 | 0.20 | 0.40 | 0.60 | 0.80 | 0.21 | 3.99 | 210 |
| 222123 | 12 | 0.48 | 0.26 | 0.1 | 1 | 0.1 | 0.30 | 0.50 | 0.70 | 0.90 | 0.16 | 4.06 | 210 |
| 133113 | 12 | 0.40 | 0.28 | 0.1 | 1 | 0.1 | 0.10 | 0.40 | 0.60 | 0.80 | 0.28 | 3.84 | 211 |
| 312222 | 12 | 0.44 | 0.27 | 0.1 | 1 | 0.1 | 0.20 | 0.40 | 0.60 | 0.89 | 0.18 | 4.00 | 212 |
| 221331 | 12 | 0.43 | 0.27 | 0.1 | 1 | 0.1 | 0.20 | 0.40 | 0.60 | 0.80 | 0.21 | 3.99 | 210 |
| 132132 | 12 | 0.38 | 0.26 | 0.1 | 1 | 0.1 | 0.20 | 0.30 | 0.60 | 0.80 | 0.22 | 3.88 | 211 |
| 323121 | 12 | 0.50 | 0.27 | 0.1 | 1 | 0.1 | 0.30 | 0.50 | 0.70 | 0.80 | 0.15 | 3.90 | 208 |
| 231322 | 13 | 0.47 | 0.28 | 0.1 | 1 | 0.1 | 0.20 | 0.50 | 0.70 | 0.90 | 0.20 | 4.06 | 210 |
| 112333 | 13 | 0.43 | 0.28 | 0.1 | 1 | 0.1 | 0.20 | 0.40 | 0.60 | 0.90 | 0.21 | 4.08 | 212 |
| 233221 | 13 | 0.51 | 0.28 | 0.1 | 1 | 0.1 | 0.30 | 0.50 | 0.70 | 0.90 | 0.14 | 4.01 | 209 |
| 123322 | 13 | 0.45 | 0.26 | 0.1 | 1 | 0.1 | 0.20 | 0.45 | 0.60 | 0.90 | 0.16 | 3.98 | 210 |
| 321223 | 13 | 0.46 | 0.29 | 0.1 | 1 | 0.1 | 0.20 | 0.45 | 0.70 | 0.90 | 0.20 | 3.95 | 212 |
| 131233 | 13 | 0.41 | 0.27 | 0.1 | 1 | 0.1 | 0.20 | 0.40 | 0.60 | 0.80 | 0.24 | 3.95 | 209 |
| 313133 | 14 | 0.48 | 0.29 | 0.1 | 1 | 0.1 | 0.20 | 0.50 | 0.70 | 0.90 | 0.21 | 4.03 | 209 |
| 331331 | 14 | 0.47 | 0.28 | 0.1 | 1 | 0.1 | 0.20 | 0.50 | 0.70 | 0.90 | 0.16 | 4.06 | 210 |
| 332231 | 14 | 0.55 | 0.26 | 0.1 | 1 | 0.11 | 0.30 | 0.60 | 0.70 | 0.90 | 0.10 | 4.07 | 212 |
| 223232 | 14 | 0.52 | 0.26 | 0.1 | 1 | 0.1 | 0.30 | 0.50 | 0.70 | 0.90 | 0.12 | 4.05 | 211 |
| 322233* | 15 | 0.56 | 0.23 | 0.2 | 0.9 | 0.2 | 0.30 | 0.60 | 0.80 | 0.90 | 0.11 | 6.65 | 211 |
| 222333* | 15 | 0.53 | 0.22 | 0.2 | 0.9 | 0.2 | 0.30 | 0.50 | 0.70 | 0.80 | 0.13 | 6.28 | 213 |
| 223323* | 15 | 0.54 | 0.23 | 0.2 | 0.9 | 0.2 | 0.30 | 0.50 | 0.70 | 0.90 | 0.14 | 6.23 | 211 |
| 332232* | 15 | 0.58 | 0.22 | 0.2 | 0.9 | 0.2 | 0.40 | 0.60 | 0.80 | 0.90 | 0.11 | 6.57 | 236 |
| 233232* | 15 | 0.52 | 0.21 | 0.2 | 0.9 | 0.2 | 0.40 | 0.50 | 0.70 | 0.80 | 0.12 | 6.25 | 213 |
| 332313 | 15 | 0.53 | 0.26 | 0.1 | 1 | 0.1 | 0.30 | 0.50 | 0.70 | 0.90 | 0.12 | 4.03 | 213 |
| 323223* | 15 | 0.55 | 0.23 | 0.2 | 0.9 | 0.2 | 0.40 | 0.50 | 0.80 | 0.90 | 0.14 | 6.45 | 212 |
| 232233* | 15 | 0.54 | 0.21 | 0.2 | 0.9 | 0.2 | 0.40 | 0.60 | 0.70 | 0.80 | 0.11 | 6.29 | 210 |
| 333222* | 15 | 0.55 | 0.23 | 0.2 | 0.9 | 0.2 | 0.40 | 0.60 | 0.70 | 0.90 | 0.16 | 6.23 | 188 |
| **Included TTO data only** | | | | | | | |  |  |  |  |  |  |
| **Config** | **LSS** | **Mean** | **Stdev** | **Min** | **Max** | **10%** | **25%** | **50%** | **75%** | **90%** | **% lowest** | **Mean its** | **N evals** |
| 111111 | 6 | 0.22 | 0.17 | 0.1 | 0.9 | 0.1 | 0.10 | 0.20 | 0.30 | 0.50 | 0.49 | 4.07 | 1344 |
| 111121 | 7 | 0.28 | 0.21 | 0.1 | 0.9 | 0.1 | 0.10 | 0.20 | 0.40 | 0.60 | 0.40 | 4.07 | 168 |
| 122211 | 9 | 0.33 | 0.26 | 0.1 | 1 | 0.1 | 0.10 | 0.30 | 0.50 | 0.70 | 0.39 | 3.92 | 175 |
| 211113 | 9 | 0.36 | 0.26 | 0.1 | 1 | 0.1 | 0.10 | 0.30 | 0.50 | 0.80 | 0.30 | 3.95 | 175 |
| 321112 | 10 | 0.41 | 0.27 | 0.1 | 1 | 0.1 | 0.20 | 0.40 | 0.60 | 0.80 | 0.24 | 3.94 | 163 |
| 113212 | 10 | 0.32 | 0.21 | 0.1 | 0.9 | 0.1 | 0.10 | 0.30 | 0.50 | 0.60 | 0.28 | 4.04 | 167 |
| 213311 | 11 | 0.37 | 0.26 | 0.1 | 1 | 0.1 | 0.10 | 0.30 | 0.50 | 0.76 | 0.27 | 3.89 | 175 |
| 212312 | 11 | 0.41 | 0.26 | 0.1 | 1 | 0.1 | 0.20 | 0.40 | 0.60 | 0.80 | 0.22 | 4.02 | 168 |
| 222123 | 12 | 0.48 | 0.26 | 0.1 | 1 | 0.1 | 0.30 | 0.50 | 0.70 | 0.80 | 0.15 | 4.08 | 167 |
| 132132 | 12 | 0.37 | 0.25 | 0.1 | 0.9 | 0.1 | 0.20 | 0.30 | 0.60 | 0.80 | 0.24 | 3.87 | 174 |
| 312222 | 12 | 0.43 | 0.26 | 0.1 | 1 | 0.1 | 0.20 | 0.40 | 0.60 | 0.80 | 0.17 | 4.08 | 157 |
| 133113 | 12 | 0.38 | 0.27 | 0.1 | 1 | 0.1 | 0.10 | 0.30 | 0.60 | 0.80 | 0.33 | 3.83 | 156 |
| 221331 | 12 | 0.40 | 0.26 | 0.1 | 1 | 0.1 | 0.20 | 0.40 | 0.60 | 0.80 | 0.24 | 3.97 | 156 |
| 323121 | 12 | 0.51 | 0.27 | 0.1 | 1 | 0.1 | 0.30 | 0.50 | 0.70 | 0.80 | 0.13 | 3.95 | 166 |
| 112333 | 13 | 0.40 | 0.26 | 0.1 | 1 | 0.1 | 0.20 | 0.40 | 0.60 | 0.80 | 0.24 | 3.95 | 164 |
| 233221 | 13 | 0.51 | 0.28 | 0.1 | 1 | 0.1 | 0.30 | 0.50 | 0.70 | 0.90 | 0.15 | 4.06 | 162 |
| 231322 | 13 | 0.47 | 0.28 | 0.1 | 1 | 0.1 | 0.20 | 0.50 | 0.70 | 0.90 | 0.18 | 4.11 | 175 |
| 123322 | 13 | 0.43 | 0.25 | 0.1 | 1 | 0.1 | 0.20 | 0.40 | 0.60 | 0.78 | 0.17 | 3.88 | 173 |
| 321223 | 13 | 0.45 | 0.28 | 0.1 | 1 | 0.1 | 0.20 | 0.40 | 0.70 | 0.90 | 0.21 | 3.94 | 175 |
| 131233 | 13 | 0.39 | 0.27 | 0.1 | 1 | 0.1 | 0.10 | 0.40 | 0.60 | 0.80 | 0.26 | 3.99 | 167 |
| 331331 | 14 | 0.45 | 0.26 | 0.1 | 1 | 0.1 | 0.20 | 0.40 | 0.60 | 0.80 | 0.15 | 4.16 | 167 |
| 313133 | 14 | 0.48 | 0.29 | 0.1 | 1 | 0.1 | 0.20 | 0.50 | 0.70 | 0.80 | 0.21 | 4.01 | 174 |
| 332231 | 14 | 0.55 | 0.26 | 0.1 | 1 | 0.14 | 0.35 | 0.60 | 0.70 | 0.90 | 0.10 | 3.96 | 175 |
| 223232 | 14 | 0.53 | 0.26 | 0.1 | 1 | 0.1 | 0.30 | 0.50 | 0.72 | 0.90 | 0.11 | 4.10 | 168 |
| 222333* | 15 | 0.55 | 0.22 | 0.2 | 0.9 | 0.3 | 0.40 | 0.60 | 0.70 | 0.86 | 0.10 | 6.16 | 165 |
| 223323* | 15 | 0.55 | 0.22 | 0.2 | 0.9 | 0.2 | 0.40 | 0.60 | 0.70 | 0.80 | 0.12 | 6.32 | 168 |
| 322233* | 15 | 0.58 | 0.23 | 0.2 | 0.9 | 0.3 | 0.40 | 0.60 | 0.80 | 0.90 | 0.09 | 6.66 | 176 |
| 332232* | 15 | 0.59 | 0.21 | 0.2 | 0.9 | 0.3 | 0.40 | 0.60 | 0.80 | 0.90 | 0.08 | 6.53 | 190 |
| 233232* | 15 | 0.55 | 0.21 | 0.2 | 0.9 | 0.3 | 0.40 | 0.60 | 0.70 | 0.80 | 0.09 | 6.16 | 170 |
| 332313 | 15 | 0.54 | 0.26 | 0.1 | 1 | 0.1 | 0.30 | 0.60 | 0.70 | 0.90 | 0.11 | 4.06 | 170 |
| 323223* | 15 | 0.57 | 0.23 | 0.2 | 0.9 | 0.24 | 0.40 | 0.60 | 0.80 | 0.90 | 0.10 | 6.45 | 175 |
| 232233* | 15 | 0.55 | 0.21 | 0.2 | 0.9 | 0.2 | 0.40 | 0.60 | 0.70 | 0.80 | 0.12 | 6.15 | 168 |
| 333222* | 15 | 0.57 | 0.23 | 0.2 | 0.9 | 0.2 | 0.40 | 0.60 | 0.80 | 0.90 | 0.15 | 6.16 | 142 |

Table S5. Descriptive statistics for TTO answers per state. All TTO data on the top and TTO data after excluding participants according to the combined inconsistency score on the bottom. ‘% at lowest’ indicates the % of valuations at the lowest possible value (0.2 for learning states, 0.1 otherwise).
Learning state configs are marked with *. Mean iterations (Mean its). Number of evaluations (N evals). Level sum score (LSS). Time trade-off (TTO). Standard deviation (Stdev).


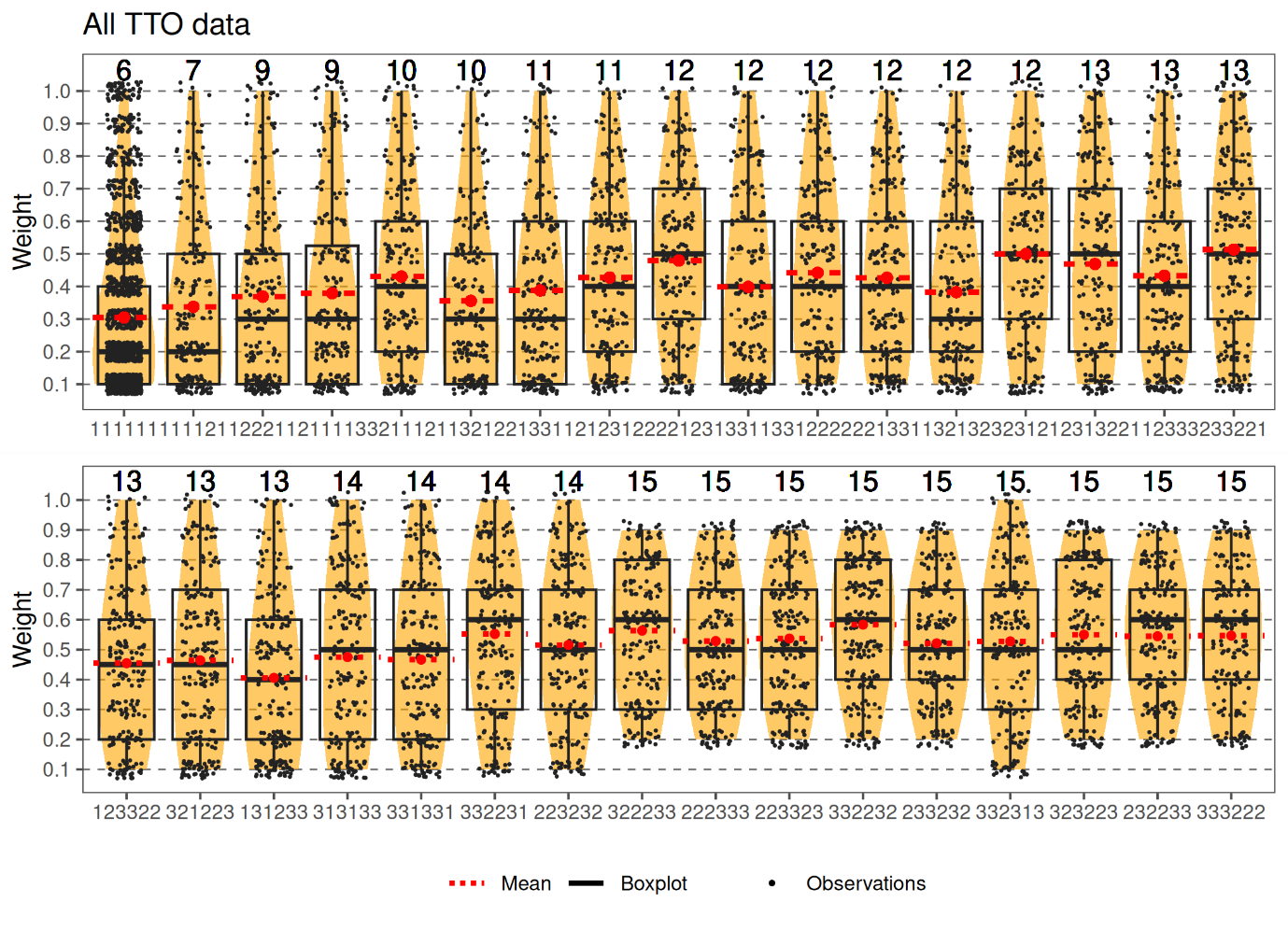


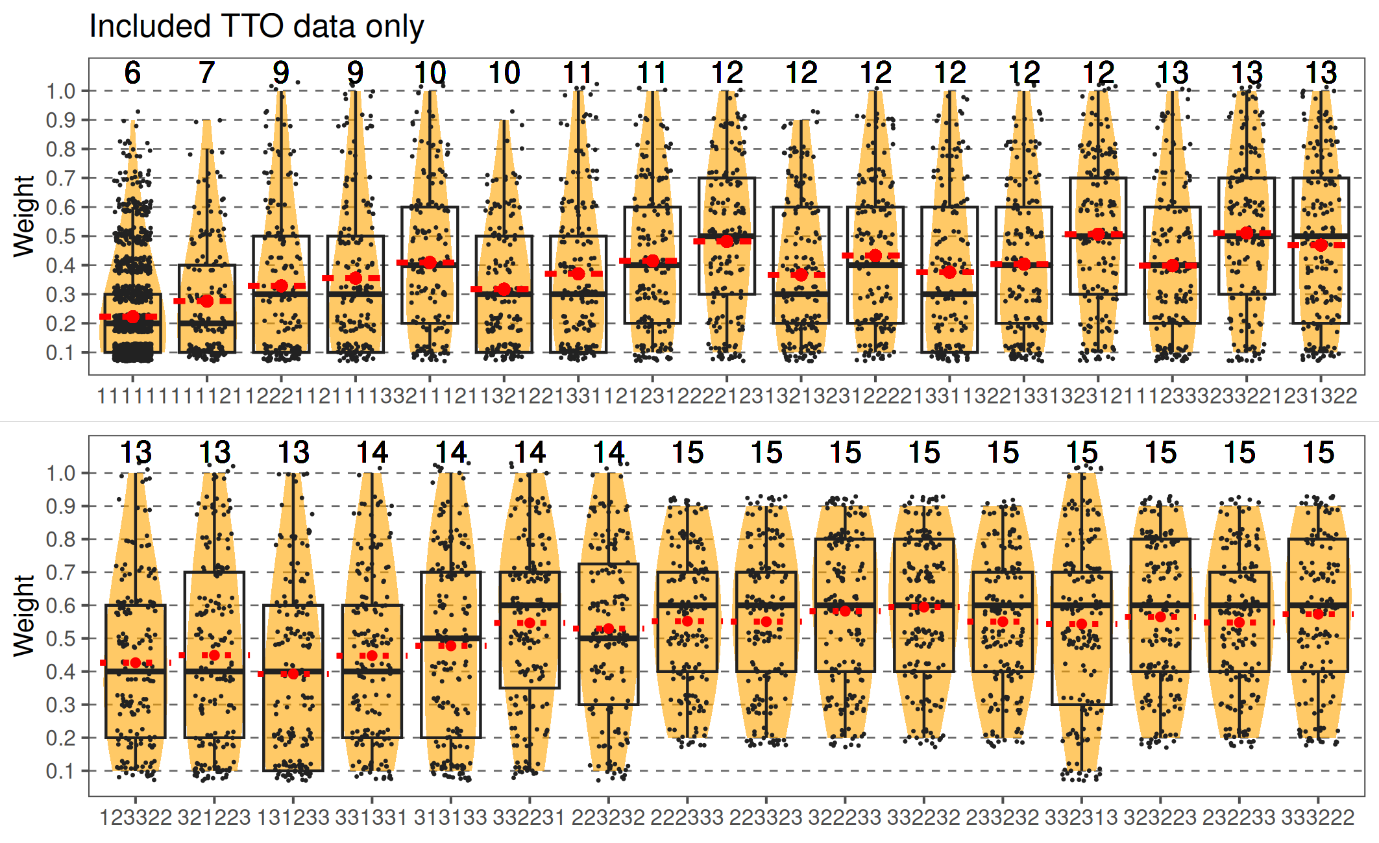


Fig S5. TTO answers per state. Top two rows contain all TTO answers, the bottom two rows only TTO data after exclusion of 20% of participants with worse combined inconsistency scores. Depicted are violin plot of distribution density, with overlaid jittered corresponding to individual TTO answers. For the boxplot, middle lines correspond to the median, and hinges correspond to first and 3^rd^ quartiles. The whiskers correspond to the interquartile range times 1.5. Numbers on top are the level sum scores. The red dotted horizontal lines represent the mean values. Time trade-off (TTO).


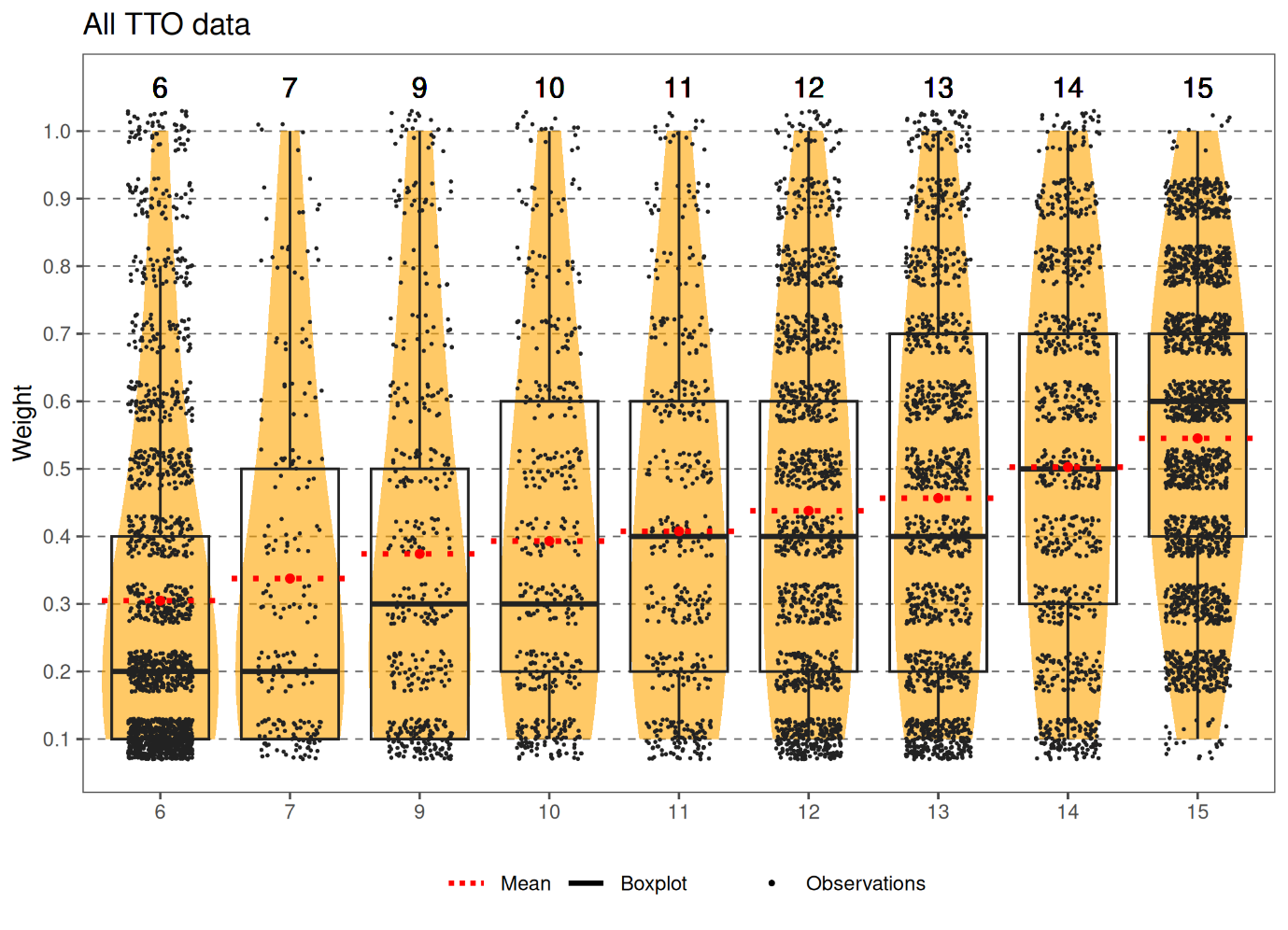


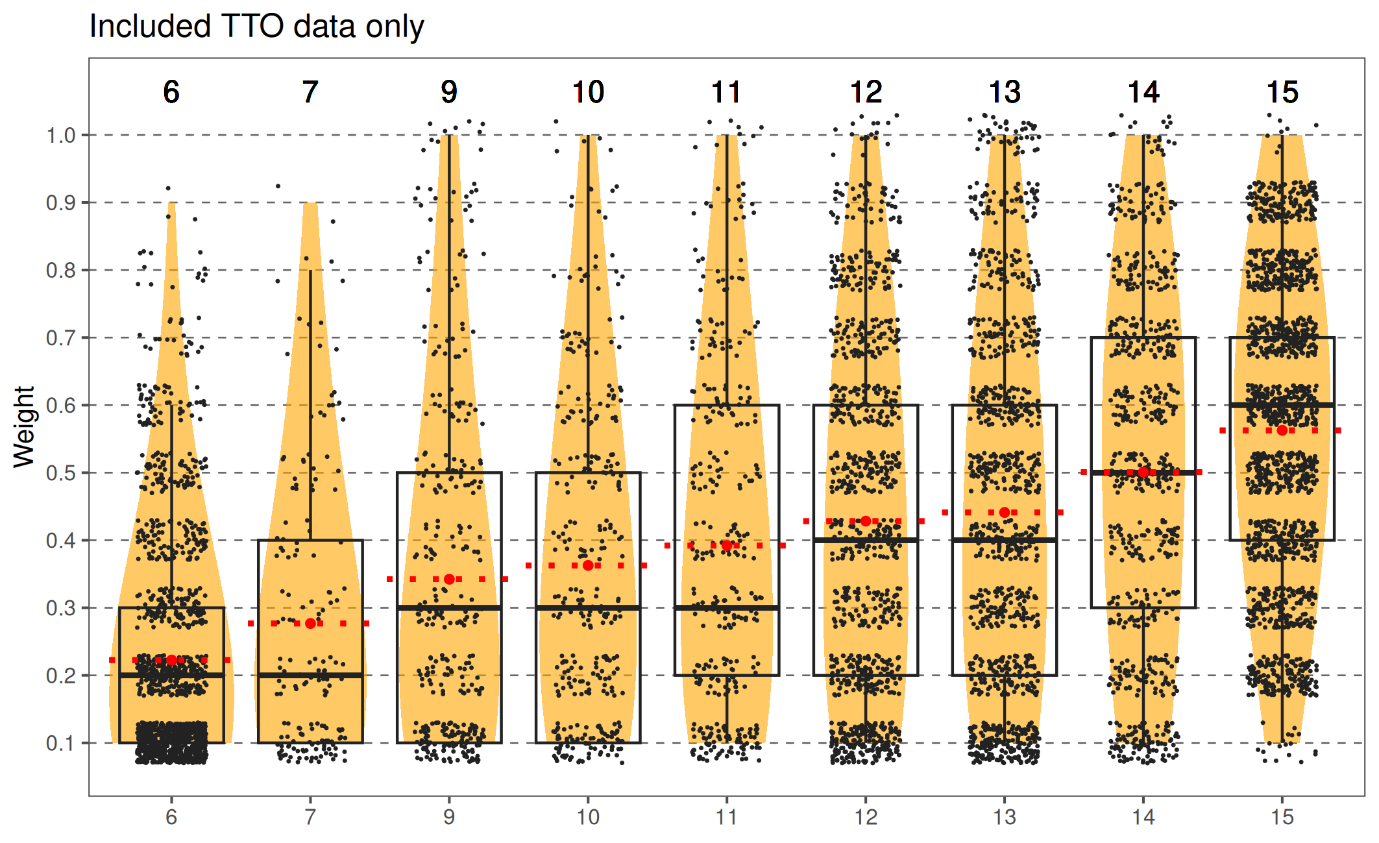


Fig S6. TTO answers per LLS. The top row contains all TTO answers, the bottom row only TTO data after exclusion of 20% of participants with worse combined inconsistency scores. Depicted are violinplots of distribution density, with overlaid jittered corresponding to individual TTO answers. For the boxplot, middle lines correspond to the median, and hinges correspond to first and 3^rd^ quartiles. The whiskers correspond to the interquartile range times 1.5. Numbers on top are the LLS. The red dotted horizontal lines represent the mean values. Level sum scores (LLS). Time trade-off (TTO).

# Inconsistencies

- 1. Definition

Some states’ configuration dominate other state configurations, for example 333333 dominates 111111 and 222222 because all attribute levels are higher. If that is not reflected in the TTO answers an inconsistency occurs.

For the TTO answers of a participant for any two TTO states in the participants block, a strict inconsistency is defined as:

- All levels of state 1 are higher or equal and at least one is higher than the levels of state 2 but the TTO answers for state 1 is *lower* than for state 2

And a weak inconsistency as:

- All levels of state 1 are higher or equal and at least one is higher than the levels of state 2 but the TTO answers for state 1 is *lower or equal* than for state 2
  1. Distribution

|  | **All data** |  |  | **Included TTO data only** |  |  |
| --- | --- | --- | --- | --- | --- | --- |
| **Type** | **N inconsistencies** | **Percent** | **N participants** | **N inconsistencies** | **Percent** | **N participants** |
| Weak | 0 | 16.4 | 278 | 0 | 20.5 | 278 |
| Weak | 2 | 26.4 | 447 | 2 | 33 | 447 |
| Weak | 3 | 16 | 271 | 3 | 19.6 | 265 |
| Weak | 4 | 24.1 | 409 | 4 | 21.9 | 297 |
| Weak | 5 | 17.1 | 289 | 5 | 4.9 | 67 |
| Strict | 0 | 50 | 847 | 0 | 61.7 | 835 |
| Strict | 2 | 23.7 | 402 | 2 | 27.3 | 370 |
| Strict | 3 | 8.1 | 137 | 3 | 6 | 81 |
| Strict | 4 | 11 | 187 | 4 | 4.3 | 58 |
| Strict | 5 | 7.1 | 121 | 5 | 0.7 | 10 |

Table S6. Distribution of inconsistencies. The minimum number of inconsistencies to occur is two as at a minimum two TTO answers need are needed to be inconsistent to each other.

# Combined inconsistency severity (CIS) score

- 1. Calculation

The *severity* of an inconsistency was defined as ($i$: index over attributes, $j$, $k$, $j\neq k$: indexes for two TTO states form the block from participant $p$):

$$L_{pjk}=\sum_{i} \left| S_{ji}-S_{ki} \right|$$

$$W_{pjk}=\left| w_{j}-w_{k} \right|$$

with $W$ being the absolute difference of the TTO answers and $L$ the absolute difference in TTO level attributes for the two involved states.

Those scores where then normalized to [0,1]:

$$Lnorm_{pjk}=\frac{L_{pjk}-min\left( L_{pjk} \right)}{max\left( L_{pjk} \right)-min\left( L_{pjk} \right)}$$

$$Wnorm_{pjk}=\frac{W_{pjk}-min\left( W_{pjk} \right)}{max\left( W_{pjk} \right)-min\left( W_{pjk} \right)}$$

And then summed for each participant $p$ as an approximation of data quality per participant:

$$CISscore_{p}=\left( \sum_{jk} Lnorm_{pjk}+\sum_{jk} Wnorm_{pjk} \right)$$

Lastly, we calculated the score percentiles for participant per block to enable the exclusion of specific proportions of data:

$$CISpercentilescore_{p}=\frac{rank_{block}\left( score_{p} \right)}{n_{block}}$$

- 1. Distribution CIS score


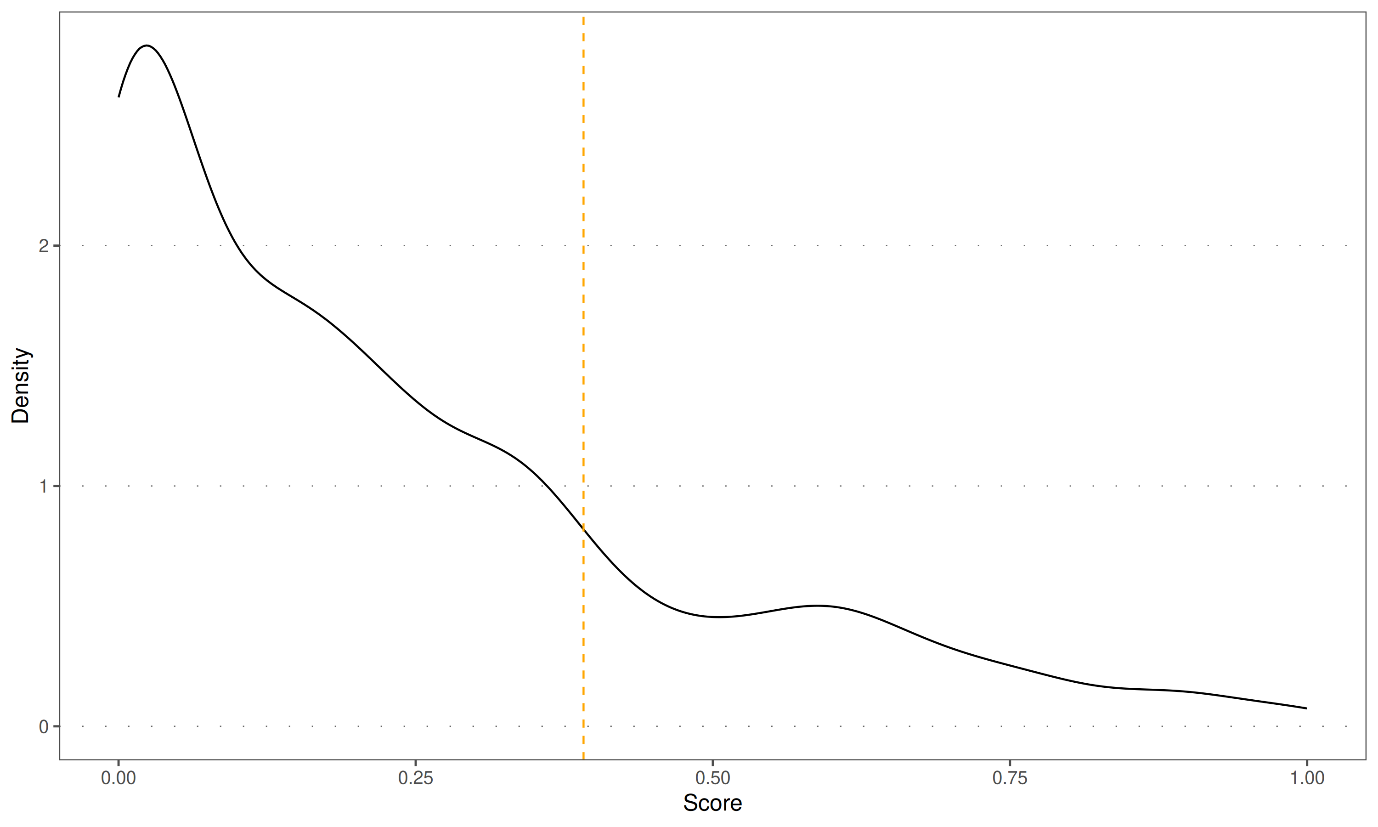


Fig S7. Density plot of the combined inconsistency severity (CIS) score. The horizontal dashed line indicates the 80% cut-off point.

For example, a participant right above the cut-off score answered:

0.7 for 222333 (learning state),
0.1 for 233221,
0.1 for 321112,
0.7 for 112333,
and 0.6 for 111111.

This results in 3 inconsistencies of 112333 and 222333 (both valued at weight 0.7), 111111 and 233221 (0.6 vs 0.1), and 11111 and 321112 (0.6 vs 0.1).

# Model specifications

The variable coding scheme for TTO models consisted of a constant corresponding to 111111 (hereinafter *pit* state), a dummy for the difference between level 1 and 2 and one for the difference between 2 and 3 for each dimension. With this coding, inconsistent coefficients would show up negative and be easily spotted, and the proportion between coefficients for levels is readily interpretable.

We analysed the DCE data with a conditional logit model and the same coding approach where the coefficients stand for the difference between levels 1 and 2, and level 2 and 3 respectively. Instead of modelling absolute values, we specified the model matrix to encode the latent differences between the attribute levels of the two states. For example, preferring 111111 over 333333 would result in the regression row o1~mu + (0, -1|0, -1|0,-1|0,-1|0, -1|0,-1) * B (comma separating attribute levels and vertical dashes separating attributes). We used medians for Bayesian point estimates.

- 1. Stan code final model

data **{**

**int<**lower**=**0**>** K**;** // number of predictors

// number of groups for random intercept

**int<**lower**=**0**>** J**;**

// number of observations per interval type

**int<**lower**=**0**>** N_rc**;**

**int<**lower**=**0**>** N_lc**;**

**int<**lower**=**0**>** N_pt**;**

// model matix per interval type

**matrix[**N_rc**,** K**]** x_rc**;**

**matrix[**N_lc**,** K**]** x_lc**;**

**matrix[**N_pt**,** K**]** x_pt**;**

// outcomes per interval group

**vector[**N_rc**]** y_rc**;**

**vector[**N_lc**]** y_lc**;**

**vector[**N_pt**]** y_pt**;**

// group indexes specific to each interval group

array **[**N_rc**]** **int<**lower**=**1**,** upper**=**J**>** intercept_group_rc**;**

array **[**N_lc**]** **int<**lower**=**1**,** upper**=**J**>** intercept_group_lc**;**

array **[**N_pt**]** **int<**lower**=**1**,** upper**=**J**>** intercept_group_pt**;**

// number of logit observations

**int<**lower**=**0**>** N_prob**;**

// logit model matrix

**matrix[**N_prob**,** K**]** x_prob**;**

array**[**N_prob**]** **int<**lower**=**0**,**upper**=**1**>** y_prob**;**

// config to result in value highest config if multiplied by X * anchor factor

**matrix[**1**,** K**]** x_mound**;**

**}**

parameters **{**

// coefficients

**vector<**lower**=**0**>[**K**]** b_com**;**

// varying intercepts

**vector[**J**]** u_raw**;**

// standard deviation errors

**real<**lower**=**0**>** sigma_e**;**

// standard deviation varying intercept

**real<**lower**=**0**>** sigma_u**;**

// constant

**real** **<**lower**=**0**>**mu**;**

// parameter that scale the coefficients for logit

**real<**lower**=**0**>** scale_prob**;**

// parameter to anchor coefficients on [0,1] scale

**real** af**;**

**}**

transformed parameters**{**

// Non-centered parametrization for varing intercept

// https://mc-stan.org/docs/stan-users-guide/reparameterization.html

**vector[**J**]** alpha**;**

alpha **=** sigma_u ***** u_raw**;**

**}**

model **{**

// priors

sigma_u **~** **normal(**0**,**2**);**

sigma_e **~** **normal(**0**,**2**);**

mu **~** **normal(**0**,** 2**);**

u_raw **~** std_normal**();**

// logit

**target** **+=** bernoulli_logit_lpmf**(**y_prob**|** x_prob ***** b_com ***** scale_prob **);**

// point

y_pt**~** **normal(** alpha**[**intercept_group_pt**]** **+** mu**+** x_pt ***** b_com ***** af**,** sigma_e**);**

// left censored

**for(**i **in** 1**:**N_lc**){**

**target** **+=** normal_lcdf**(**y_lc**[**i**]** **|** alpha**[**intercept_group_pt**[**i**]]+**mu**+** x_lc**[**i**,]***b_com*****af**,** sigma_e**);**

**}**

// right censored

**for(**i **in** 1**:**N_rc**){**

**target** **+=** normal_lccdf**(**y_rc**[**i**]** **|** alpha**[**intercept_group_pt**[**i**]]** **+** mu**+** x_rc**[**i**,]***b_com*****af**,** sigma_e**);**

**}**

// tight prior sd to anchor b_com on [0,1]

1 **~** **normal(**mu**+**x_mound*****b_com**,** 0.01**);**

**}**

# Extended model results

|  | **MAE** | | **Accuracy (%)** | | **CI width weights (%)** | | **DCE logit fidelity** | | **Mean rank** | **111111** | **Diff 333333 to 1** | **ELPD LOO** | **Range** |
| --- | --- | --- | --- | --- | --- | --- | --- | --- | --- | --- | --- | --- | --- |
| **Model** | **Rank** | **Value** | **Rank** | **Value** | **Rank** | **Value** | **Rank** | **Value** |  |  |  |  |  |
| Hybrid anchor scale, censoring, het. | 98.9 | 0.195 | 100 | 73.3 | 87.9 | 4.85 | 100 | 3.58 | **96.7** | 0.114 | 0.00 | -8526.63 | 0.887 |
| Hybrid anchor scale, censoring | 98.9 | 0.195 | 100 | 73.3 | 87.4 | 4.89 | 99.9 | 3.66 | **96.6** | 0.114 | 0.00 | -8526.84 | 0.887 |
| Hybrid anchor scale | 97.1 | 0.196 | 100 | 73.3 | 87.2 | 4.91 | 99.9 | 3.71 | **96.1** | 0.216 | 0.00 | -5623.26 | 0.786 |
| TTO linear anchor scale, censoring, VI | 100 | 0.194 | 83.4 | 72.2 | 0 | 13.1 | 78.6 | 29.6 | **65.5** | 0.128 | 0.00 | -3022.54 | 0.873 |
| Hybrid discapability, censoring, het. | 52.2 | 0.23 | 50.8 | 69.8 | 100 | 3.71 | 52 | 61.9 | **63.8** | -0.0277 | 0.00 | -9218.51 | 1.03 |
| Hybrid discapability, censoring, VI | 49.9 | 0.232 | 48.8 | 69.7 | 97 | 3.99 | 49.7 | 64.6 | **61.3** | -0.0391 | 0.00 | -9227.26 | 1.04 |
| TTO linear discapability, censoring, VI | 65.7 | 0.22 | 32.5 | 68.5 | 80.6 | 5.53 | 9.84 | 113 | **47.2** | 0.269 | 0.00 | -28939907.77 | 0.731 |
| TTO linear discapability | 86 | 0.205 | 0 | 66.2 | 90.2 | 4.62 | 0 | 125 | **44.1** | 0.178 | 0.00 | -367.85 | 0.822 |
| Hybrid discapability | 0 | 0.27 | 53.4 | 70 | 22.1 | 11 | 53.3 | 60.3 | **32.2** | 0.366 | 0.00 | -5002.02 | 0.634 |
| Hybrid |  | 0.196 |  | 73.3 |  | 4.76 |  | 3.68 |  | 0.215 | 0.39 | -5623.35 | 0.393 |
| Hybrid censoring |  | 0.195 |  | 73.3 |  | 4.98 |  | 3.6 |  | 0.113 | 0.40 | -8526.78 | 0.486 |
| TTO linear het. |  | 0.196 |  | 72.1 |  | 11.8 |  | 29.9 |  | 0.215 | 0.40 | 152.33 | 0.38 |
| TTO linear censoring |  | 0.195 |  | 72.1 |  | 12.5 |  | 30.2 |  | 0.112 | 0.41 | -2752.66 | 0.474 |
| TTO linear censoring, VI |  | 0.194 |  | 72.2 |  | 12.2 |  | 28.7 |  | 0.126 | 0.41 | -3021.21 | 0.468 |
| TTO linear VI |  | 0.196 |  | 72.2 |  | 9.91 |  | 30.1 |  | 0.214 | 0.40 | 1553.14 | 0.381 |

Table S7. Model ranking overview table.
**Mean CI widths** given in percent of the range between 111111 and 333333 as the different model features influence the range of weights and precision relative to the covered range is more meaningful.
**Accuracy** is the mean kfold proportion of correctly predicted DCE choices.
**MAE** is the mean kfold absolute error between predicted and observed TTO answers. For anchor scale models, the anchor scale was applied before comparison with the observed TTO answers.
**DCE Logit fidelity** is the absolute sum of per-coefficient differences to the coefficients of the logit model, after rescaling of the coefficients of both models so that their sum corresponded to 100 percent.
Mean ranks takes only the columns left of it into account. The columns at the right contain additional information that is not always comparable between different model types. Shades of green in the background indicate the rank of the value, the worse the rank the lighter the shade. Model in the rows in the bottom part do not result in 333333 anchored to 1 and are thus not ranked. Ranks correspond to linear percentile ranks with 0% corresponding to the worst value and 100% to the best value.
Heteroskedasticity (het). Varying intercept (VI). Mean absolute error (MAE), discrete choice experiment (DCE) , time trade-off (TTO), credible interval (CI), expected log pointwise density (ELPD), leave-one-out (LOO).

# Predicted vs observed weights


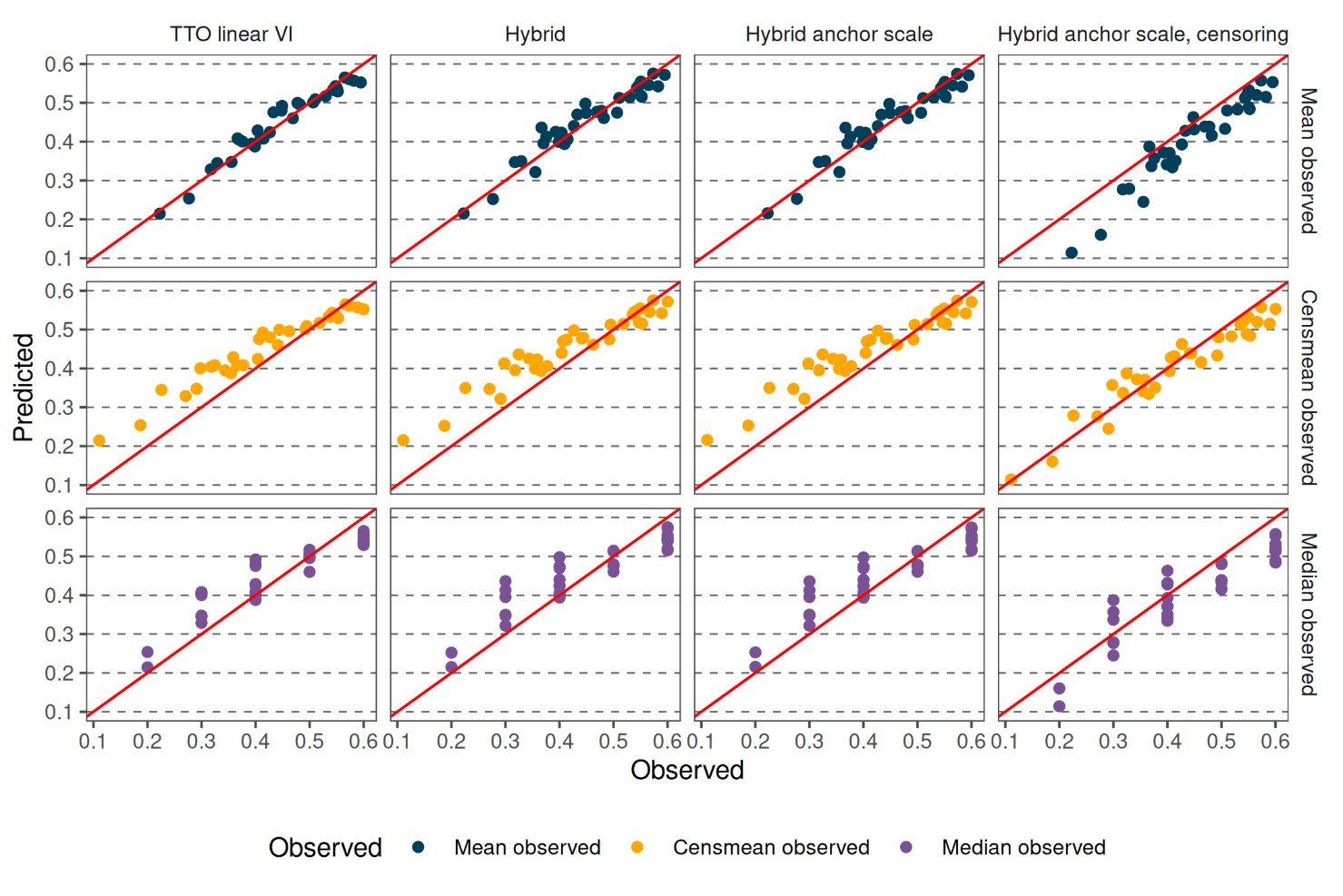


Fig S8. Predicted weights plotted against observed weights (mean, means calculated taking into account censoring, and medians) for states used in TTO questions for four selected models.


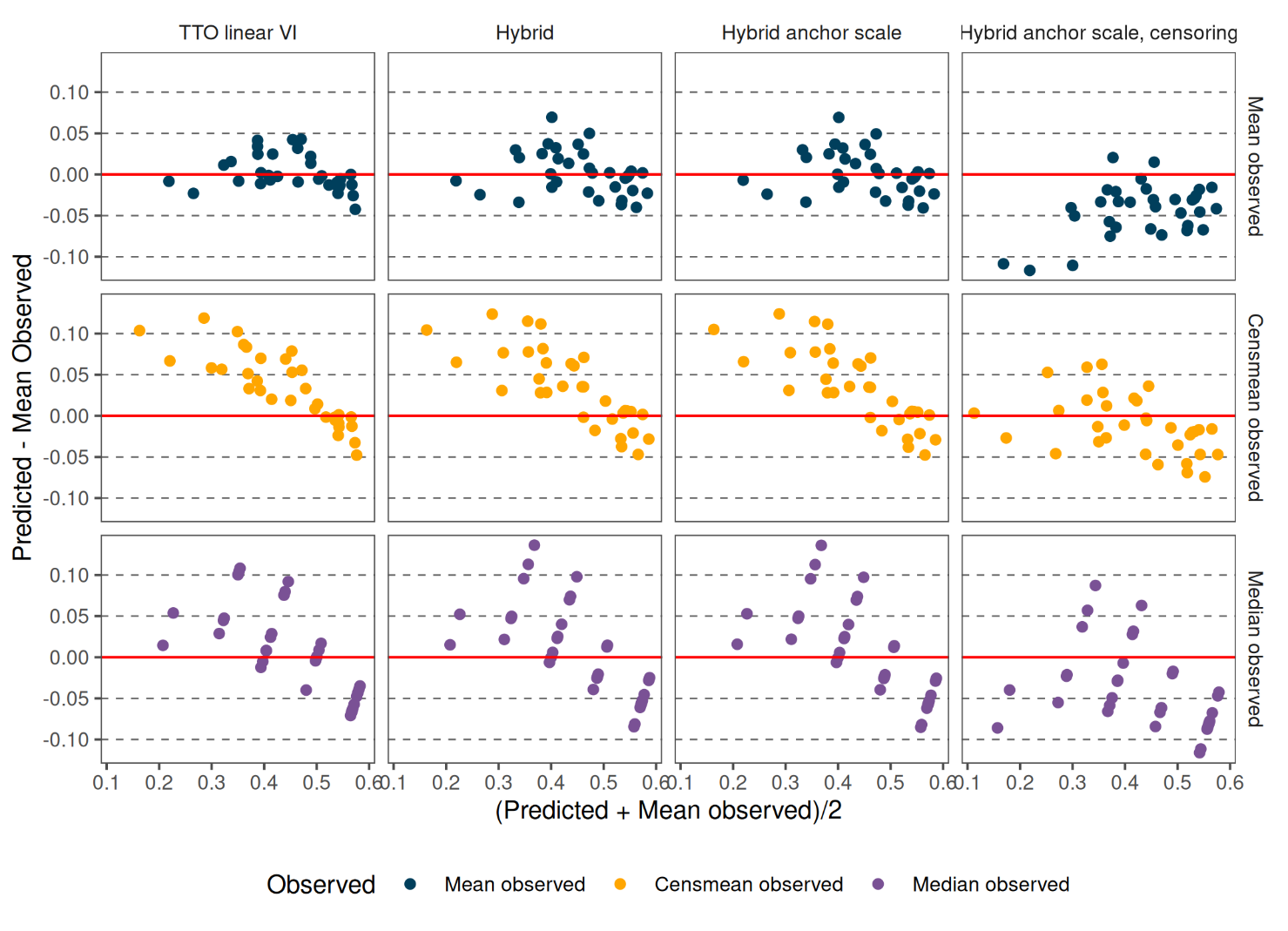


Fig S9. Bland-Altman plots of means, means taking into account censoring, and medians of observed weights and predicted weights for states used in TTO questions for four selected models. Y coordinates represent the difference of observed and predicted weights, and for X coordinates the average of predicted and mean observed weights.

# Results sensitivity analyses

| **% included TTO data** | **50%** | **60%** | **70%** | **80%** | **90%** | **100%** |
| --- | --- | --- | --- | --- | --- | --- |
| Don't agree (constant) | 0.139 ( 0.12, 0.15) | 0.122 ( 0.11, 0.14) | 0.117 ( 0.1 , 0.13) | 0.114 ( 0.1 , 0.13) | 0.156 ( 0.14, 0.17) | 0.219 ( 0.2 , 0.23) |
|  |  |  |  |  |  |  |
| *Health* | Rank 1 | Rank 1 | Rank 1 | Rank 1 | Rank 1 | Rank 1 |
| Agree partially | 0.15 ( 0.14, 0.16) | 0.153 ( 0.14, 0.16) | 0.154 ( 0.14, 0.17) | 0.154 ( 0.14, 0.17) | 0.146 ( 0.13, 0.16) | 0.134 ( 0.12, 0.14) |
| Agree completely | 0.075 ( 0.07, 0.09) | 0.076 ( 0.07, 0.09) | 0.077 ( 0.07, 0.09) | 0.078 ( 0.07, 0.09) | 0.075 ( 0.06, 0.09) | 0.07 ( 0.06, 0.08) |
|  |  |  |  |  |  |  |
| *Social relations* | Rank 2 | Rank 2 | Rank 2 | Rank 2 | Rank 2 | Rank 2 |
| Agree partially | 0.105 ( 0.09, 0.11) | 0.106 ( 0.1 , 0.12) | 0.108 ( 0.1 , 0.12) | 0.108 ( 0.1 , 0.12) | 0.102 ( 0.09, 0.11) | 0.094 ( 0.08, 0.1 ) |
| Agree completely | 0.088 ( 0.08, 0.1 ) | 0.092 ( 0.08, 0.1 ) | 0.09 ( 0.08, 0.1 ) | 0.091 ( 0.08, 0.1 ) | 0.087 ( 0.08, 0.1 ) | 0.082 ( 0.07, 0.09) |
|  |  |  |  |  |  |  |
| *Finance & housing* | Rank 3 | Rank 3 | Rank 3 | Rank 3 | Rank 3 | Rank 3 |
| Agree partially | 0.117 ( 0.11, 0.13) | 0.119 ( 0.11, 0.13) | 0.121 ( 0.11, 0.13) | 0.122 ( 0.11, 0.13) | 0.116 ( 0.1 , 0.13) | 0.106 ( 0.1 , 0.12) |
| Agree completely | 0.041 ( 0.03, 0.05) | 0.041 ( 0.03, 0.05) | 0.04 ( 0.03, 0.05) | 0.04 ( 0.03, 0.05) | 0.037 ( 0.03, 0.05) | 0.035 ( 0.02, 0.04) |
|  |  |  |  |  |  |  |
| *Occupation* | Rank 5 | Rank 5 | Rank 5 | Rank 5 | Rank 5 | Rank 5 |
| Agree partially | 0.067 ( 0.06, 0.08) | 0.07 ( 0.06, 0.08) | 0.071 ( 0.06, 0.08) | 0.073 ( 0.06, 0.08) | 0.07 ( 0.06, 0.08) | 0.066 ( 0.06, 0.08) |
| Agree completely | 0.025 ( 0.01, 0.04) | 0.023 ( 0.01, 0.03) | 0.023 ( 0.01, 0.03) | 0.021 ( 0.01, 0.03) | 0.019 ( 0.01, 0.03) | 0.017 ( 0.01, 0.03) |
|  |  |  |  |  |  |  |
| *Security* | Rank 4 | Rank 4 | Rank 4 | Rank 4 | Rank 4 | Rank 4 |
| Agree partially | 0.081 ( 0.07, 0.09) | 0.084 ( 0.07, 0.1 ) | 0.084 ( 0.07, 0.1 ) | 0.085 ( 0.07, 0.1 ) | 0.08 ( 0.07, 0.09) | 0.074 ( 0.06, 0.08) |
| Agree completely | 0.03 ( 0.02, 0.04) | 0.03 ( 0.02, 0.04) | 0.031 ( 0.02, 0.04) | 0.031 ( 0.02, 0.04) | 0.031 ( 0.02, 0.04) | 0.029 ( 0.02, 0.04) |
|  |  |  |  |  |  |  |
| *Political & civil rights* | Rank 6 | Rank 6 | Rank 6 | Rank 6 | Rank 6 | Rank 6 |
| Agree partially | 0.062 ( 0.05, 0.07) | 0.063 ( 0.05, 0.07) | 0.063 ( 0.05, 0.07) | 0.065 ( 0.05, 0.08) | 0.061 ( 0.05, 0.07) | 0.056 ( 0.05, 0.07) |
| Agree completely | 0.022 ( 0.01, 0.03) | 0.022 ( 0.01, 0.03) | 0.022 ( 0.01, 0.03) | 0.021 ( 0.01, 0.03) | 0.02 ( 0.01, 0.03) | 0.017 ( 0.01, 0.03) |
|  |  |  |  |  |  |  |
| Linear model SD | 0.242 ( 0.24, 0.25) | 0.263 ( 0.26, 0.27) | 0.283 ( 0.28, 0.29) | 0.302 ( 0.3 , 0.31) | 0.313 ( 0.31, 0.32) | 0.332 ( 0.33, 0.34) |
| Logit scale factor | 6.112 ( 5.76, 6.49) | 5.993 ( 5.64, 6.36) | 5.961 ( 5.63, 6.32) | 5.952 ( 5.6 , 6.32) | 6.254 ( 5.89, 6.65) | 6.758 ( 6.35, 7.2 ) |
| Anchor scale | 0.662 ( 0.64, 0.69) | 0.628 ( 0.6 , 0.65) | 0.59 ( 0.56, 0.62) | 0.545 ( 0.52, 0.57) | 0.494 ( 0.47, 0.52) | 0.421 ( 0.39, 0.45) |
|  |  |  |  |  |  |  |
| *K-fold* |  |  |  |  |  |  |
| MAE | 0.169 | 0.178 | 0.187 | 0.195 | 0.203 | 0.215 |
| MSE | 0.046 | 0.051 | 0.055 | 0.06 | 0.064 | 0.07 |
| Accuracy | 73.345 | 73.277 | 73.345 | 73.345 | 73.277 | 73.277 |
| ME % of range | 3.799 | 4.55 | 5.271 | 6.235 | 6.292 | 6.38 |
| MSE % of range | 5.29 | 5.76 | 6.28 | 6.76 | 7.53 | 8.98 |
| MAE % of range | 19.65 | 20.28 | 21.14 | 21.99 | 24.08 | 27.54 |
|  |  |  |  |  |  |  |
| *Other model properties* |  |  |  |  |  |  |
| ELPD LOO | -6484.381 | -7109.888 | -7779.924 | -8526.84 | -9093.559 | -9917.037 |
| Mean CI width weights | 0.042 | 0.043 | 0.043 | 0.043 | 0.042 | 0.04 |
| Mean CI width coefficients | 0.021 | 0.022 | 0.022 | 0.022 | 0.021 | 0.02 |
| N observations | 24546 | 25412 | 26249 | 27099 | 27948 | 28796 |
| N observations DCE | 20364 | 20364 | 20364 | 20364 | 20364 | 20364 |
| N observations TTO | 4182 | 5048 | 5885 | 6735 | 7584 | 8432 |
| Range | 0.862 | 0.88 | 0.884 | 0.887 | 0.845 | 0.782 |
| 333333 | 1.001 | 1.001 | 1.001 | 1.001 | 1.001 | 1.001 |
| 111111 | 0.139 | 0.122 | 0.117 | 0.114 | 0.156 | 0.219 |
| Mean 95% CI width weights % of range | 4.82 | 4.85 | 4.91 | 4.89 | 4.95 | 5.16 |
| Mean 95% CI width coefs % of range | 2.45 | 2.47 | 2.49 | 2.48 | 2.53 | 2.56 |

Table S8. Sensitivity analysis. For including 60%, 70%, 80%, 90%, and 100% of TTO data from participants according to CIS score and the model selected to generate the value set (Hybrid with censoring and anchor scale). 80% of TTO data was used to generate the value set. Mean absolute error (MAE), discrete choice experiment (DCE), time trade-off (TTO), credible interval (CI), expected log pointwise density (ELPD), leave-one-out (LOO).

# Weight table final model

| **State** | **Weight** | **95% Credible interval** |
| --- | --- | --- |
| **111111** | 0.114 | (0.098,0.129) |
| **211111** | 0.268 | (0.251,0.284) |
| **311111** | 0.346 | (0.330,0.362) |
| **121111** | 0.221 | (0.204,0.239) |
| **221111** | 0.376 | (0.357,0.395) |
| **321111** | 0.453 | (0.435,0.472) |
| **131111** | 0.312 | (0.297,0.328) |
| **231111** | 0.467 | (0.448,0.485) |
| **331111** | 0.544 | (0.526,0.562) |
| **112111** | 0.236 | (0.218,0.253) |
| **212111** | 0.390 | (0.371,0.409) |
| **312111** | 0.468 | (0.449,0.487) |
| **122111** | 0.343 | (0.324,0.363) |
| **222111** | 0.497 | (0.476,0.520) |
| **322111** | 0.575 | (0.554,0.597) |
| **132111** | 0.434 | (0.416,0.453) |
| **232111** | 0.588 | (0.567,0.610) |
| **332111** | 0.666 | (0.645,0.688) |
| **113111** | 0.275 | (0.259,0.292) |
| **213111** | 0.429 | (0.412,0.448) |
| **313111** | 0.507 | (0.489,0.525) |
| **123111** | 0.383 | (0.364,0.402) |
| **223111** | 0.537 | (0.517,0.558) |
| **323111** | 0.614 | (0.594,0.635) |
| **133111** | 0.474 | (0.456,0.492) |
| **233111** | 0.628 | (0.608,0.648) |
| **333111** | 0.706 | (0.686,0.726) |
| **111211** | 0.187 | (0.169,0.205) |
| **211211** | 0.341 | (0.321,0.361) |
| **311211** | 0.419 | (0.400,0.438) |
| **121211** | 0.295 | (0.275,0.314) |
| **221211** | 0.448 | (0.427,0.471) |
| **321211** | 0.526 | (0.505,0.548) |
| **131211** | 0.386 | (0.366,0.404) |
| **231211** | 0.540 | (0.518,0.562) |
| **331211** | 0.617 | (0.596,0.639) |
| **112211** | 0.309 | (0.290,0.328) |
| **212211** | 0.463 | (0.442,0.485) |
| **312211** | 0.540 | (0.520,0.562) |
| **122211** | 0.416 | (0.395,0.438) |
| **222211** | 0.570 | (0.547,0.595) |
| **322211** | 0.648 | (0.625,0.672) |
| **132211** | 0.507 | (0.487,0.528) |
| **232211** | 0.661 | (0.637,0.686) |
| **332211** | 0.739 | (0.716,0.763) |
| **113211** | 0.348 | (0.329,0.367) |
| **213211** | 0.502 | (0.482,0.524) |
| **313211** | 0.580 | (0.560,0.601) |
| **123211** | 0.456 | (0.434,0.477) |
| **223211** | 0.610 | (0.587,0.634) |
| **323211** | 0.688 | (0.665,0.711) |
| **133211** | 0.547 | (0.526,0.568) |
| **233211** | 0.701 | (0.678,0.724) |
| **333211** | 0.778 | (0.757,0.802) |
| **111311** | 0.208 | (0.191,0.224) |
| **211311** | 0.362 | (0.344,0.379) |
| **311311** | 0.439 | (0.422,0.457) |
| **121311** | 0.315 | (0.297,0.333) |
| **221311** | 0.469 | (0.449,0.489) |
| **321311** | 0.547 | (0.528,0.566) |
| **131311** | 0.406 | (0.389,0.423) |
| **231311** | 0.560 | (0.541,0.580) |
| **331311** | 0.638 | (0.619,0.657) |
| **112311** | 0.329 | (0.311,0.347) |
| **212311** | 0.483 | (0.464,0.503) |
| **312311** | 0.561 | (0.542,0.581) |
| **122311** | 0.437 | (0.417,0.457) |
| **222311** | 0.591 | (0.569,0.613) |
| **322311** | 0.669 | (0.647,0.690) |
| **132311** | 0.528 | (0.509,0.547) |
| **232311** | 0.682 | (0.660,0.704) |
| **332311** | 0.759 | (0.739,0.781) |
| **113311** | 0.369 | (0.352,0.386) |
| **213311** | 0.523 | (0.505,0.542) |
| **313311** | 0.601 | (0.583,0.619) |
| **123311** | 0.476 | (0.457,0.495) |
| **223311** | 0.630 | (0.610,0.652) |
| **323311** | 0.708 | (0.688,0.729) |
| **133311** | 0.567 | (0.549,0.585) |
| **233311** | 0.721 | (0.701,0.743) |
| **333311** | 0.799 | (0.779,0.819) |
| **111121** | 0.199 | (0.181,0.216) |
| **211121** | 0.353 | (0.333,0.372) |
| **311121** | 0.430 | (0.411,0.449) |
| **121121** | 0.306 | (0.287,0.326) |
| **221121** | 0.460 | (0.439,0.482) |
| **321121** | 0.538 | (0.517,0.559) |
| **131121** | 0.397 | (0.378,0.416) |
| **231121** | 0.551 | (0.529,0.573) |
| **331121** | 0.629 | (0.608,0.650) |
| **112121** | 0.320 | (0.300,0.340) |
| **212121** | 0.474 | (0.453,0.496) |
| **312121** | 0.552 | (0.531,0.573) |
| **122121** | 0.428 | (0.406,0.450) |
| **222121** | 0.582 | (0.558,0.607) |
| **322121** | 0.659 | (0.636,0.684) |
| **132121** | 0.519 | (0.498,0.540) |
| **232121** | 0.673 | (0.648,0.698) |
| **332121** | 0.751 | (0.727,0.775) |
| **113121** | 0.360 | (0.342,0.379) |
| **213121** | 0.514 | (0.494,0.535) |
| **313121** | 0.592 | (0.572,0.612) |
| **123121** | 0.467 | (0.447,0.488) |
| **223121** | 0.622 | (0.598,0.645) |
| **323121** | 0.699 | (0.677,0.722) |
| **133121** | 0.558 | (0.539,0.578) |
| **233121** | 0.712 | (0.690,0.735) |
| **333121** | 0.790 | (0.768,0.813) |
| **111221** | 0.271 | (0.252,0.292) |
| **211221** | 0.425 | (0.404,0.448) |
| **311221** | 0.503 | (0.482,0.525) |
| **121221** | 0.379 | (0.357,0.401) |
| **221221** | 0.533 | (0.509,0.558) |
| **321221** | 0.611 | (0.587,0.635) |
| **131221** | 0.470 | (0.449,0.492) |
| **231221** | 0.624 | (0.600,0.649) |
| **331221** | 0.702 | (0.679,0.726) |
| **112221** | 0.393 | (0.372,0.415) |
| **212221** | 0.547 | (0.524,0.572) |
| **312221** | 0.625 | (0.602,0.648) |
| **122221** | 0.501 | (0.478,0.524) |
| **222221** | 0.655 | (0.629,0.682) |
| **322221** | 0.732 | (0.707,0.758) |
| **132221** | 0.592 | (0.569,0.615) |
| **232221** | 0.746 | (0.720,0.773) |
| **332221** | 0.823 | (0.798,0.850) |
| **113221** | 0.433 | (0.413,0.453) |
| **213221** | 0.587 | (0.564,0.610) |
| **313221** | 0.664 | (0.643,0.687) |
| **123221** | 0.540 | (0.518,0.563) |
| **223221** | 0.694 | (0.669,0.720) |
| **323221** | 0.772 | (0.748,0.797) |
| **133221** | 0.631 | (0.609,0.654) |
| **233221** | 0.785 | (0.761,0.811) |
| **333221** | 0.863 | (0.839,0.889) |
| **111321** | 0.292 | (0.273,0.311) |
| **211321** | 0.446 | (0.425,0.467) |
| **311321** | 0.524 | (0.504,0.544) |
| **121321** | 0.400 | (0.379,0.421) |
| **221321** | 0.554 | (0.531,0.576) |
| **321321** | 0.631 | (0.609,0.654) |
| **131321** | 0.491 | (0.471,0.511) |
| **231321** | 0.645 | (0.623,0.667) |
| **331321** | 0.722 | (0.701,0.745) |
| **112321** | 0.414 | (0.393,0.435) |
| **212321** | 0.568 | (0.545,0.590) |
| **312321** | 0.646 | (0.623,0.667) |
| **122321** | 0.521 | (0.499,0.544) |
| **222321** | 0.675 | (0.650,0.701) |
| **322321** | 0.753 | (0.729,0.778) |
| **132321** | 0.612 | (0.591,0.634) |
| **232321** | 0.766 | (0.741,0.792) |
| **332321** | 0.844 | (0.820,0.869) |
| **113321** | 0.453 | (0.434,0.473) |
| **213321** | 0.607 | (0.587,0.629) |
| **313321** | 0.685 | (0.665,0.706) |
| **123321** | 0.561 | (0.540,0.582) |
| **223321** | 0.715 | (0.692,0.738) |
| **323321** | 0.793 | (0.770,0.816) |
| **133321** | 0.652 | (0.632,0.673) |
| **233321** | 0.806 | (0.783,0.829) |
| **333321** | 0.884 | (0.861,0.907) |
| **111131** | 0.230 | (0.212,0.247) |
| **211131** | 0.384 | (0.366,0.403) |
| **311131** | 0.462 | (0.444,0.479) |
| **121131** | 0.338 | (0.319,0.356) |
| **221131** | 0.491 | (0.472,0.512) |
| **321131** | 0.569 | (0.549,0.589) |
| **131131** | 0.429 | (0.411,0.446) |
| **231131** | 0.583 | (0.562,0.604) |
| **331131** | 0.660 | (0.641,0.680) |
| **112131** | 0.352 | (0.333,0.370) |
| **212131** | 0.506 | (0.486,0.526) |
| **312131** | 0.584 | (0.564,0.603) |
| **122131** | 0.459 | (0.439,0.480) |
| **222131** | 0.613 | (0.591,0.636) |
| **322131** | 0.691 | (0.669,0.713) |
| **132131** | 0.550 | (0.531,0.570) |
| **232131** | 0.704 | (0.682,0.727) |
| **332131** | 0.782 | (0.760,0.804) |
| **113131** | 0.391 | (0.374,0.409) |
| **213131** | 0.545 | (0.526,0.565) |
| **313131** | 0.623 | (0.604,0.642) |
| **123131** | 0.499 | (0.480,0.518) |
| **223131** | 0.653 | (0.632,0.675) |
| **323131** | 0.731 | (0.710,0.752) |
| **133131** | 0.590 | (0.571,0.608) |
| **233131** | 0.744 | (0.723,0.766) |
| **333131** | 0.822 | (0.801,0.843) |
| **111231** | 0.303 | (0.283,0.323) |
| **211231** | 0.457 | (0.436,0.479) |
| **311231** | 0.535 | (0.514,0.556) |
| **121231** | 0.411 | (0.389,0.431) |
| **221231** | 0.565 | (0.542,0.589) |
| **321231** | 0.642 | (0.620,0.665) |
| **131231** | 0.501 | (0.481,0.522) |
| **231231** | 0.655 | (0.632,0.680) |
| **331231** | 0.733 | (0.711,0.756) |
| **112231** | 0.425 | (0.404,0.445) |
| **212231** | 0.579 | (0.556,0.602) |
| **312231** | 0.656 | (0.634,0.679) |
| **122231** | 0.532 | (0.510,0.555) |
| **222231** | 0.686 | (0.662,0.712) |
| **322231** | 0.764 | (0.740,0.789) |
| **132231** | 0.623 | (0.602,0.645) |
| **232231** | 0.777 | (0.753,0.803) |
| **332231** | 0.855 | (0.831,0.880) |
| **113231** | 0.464 | (0.444,0.484) |
| **213231** | 0.618 | (0.597,0.641) |
| **313231** | 0.696 | (0.675,0.717) |
| **123231** | 0.572 | (0.550,0.594) |
| **223231** | 0.726 | (0.702,0.751) |
| **323231** | 0.803 | (0.780,0.828) |
| **133231** | 0.663 | (0.642,0.684) |
| **233231** | 0.817 | (0.793,0.842) |
| **333231** | 0.894 | (0.872,0.919) |
| **111331** | 0.324 | (0.305,0.342) |
| **211331** | 0.478 | (0.458,0.497) |
| **311331** | 0.555 | (0.536,0.574) |
| **121331** | 0.431 | (0.411,0.450) |
| **221331** | 0.585 | (0.564,0.606) |
| **321331** | 0.663 | (0.643,0.683) |
| **131331** | 0.522 | (0.504,0.541) |
| **231331** | 0.676 | (0.655,0.698) |
| **331331** | 0.754 | (0.734,0.774) |
| **112331** | 0.445 | (0.426,0.464) |
| **212331** | 0.599 | (0.579,0.621) |
| **312331** | 0.677 | (0.657,0.697) |
| **122331** | 0.553 | (0.533,0.574) |
| **222331** | 0.707 | (0.684,0.730) |
| **322331** | 0.785 | (0.763,0.807) |
| **132331** | 0.644 | (0.624,0.664) |
| **232331** | 0.798 | (0.775,0.821) |
| **332331** | 0.876 | (0.854,0.898) |
| **113331** | 0.485 | (0.467,0.503) |
| **213331** | 0.639 | (0.620,0.659) |
| **313331** | 0.717 | (0.698,0.736) |
| **123331** | 0.592 | (0.573,0.612) |
| **223331** | 0.746 | (0.725,0.769) |
| **323331** | 0.824 | (0.803,0.845) |
| **133331** | 0.683 | (0.665,0.702) |
| **233331** | 0.837 | (0.817,0.859) |
| **333331** | 0.915 | (0.895,0.937) |
| **111112** | 0.179 | (0.161,0.196) |
| **211112** | 0.333 | (0.314,0.352) |
| **311112** | 0.410 | (0.391,0.429) |
| **121112** | 0.286 | (0.267,0.305) |
| **221112** | 0.440 | (0.419,0.462) |
| **321112** | 0.518 | (0.497,0.539) |
| **131112** | 0.377 | (0.358,0.396) |
| **231112** | 0.531 | (0.510,0.553) |
| **331112** | 0.609 | (0.588,0.630) |
| **112112** | 0.300 | (0.282,0.319) |
| **212112** | 0.454 | (0.434,0.475) |
| **312112** | 0.532 | (0.512,0.553) |
| **122112** | 0.408 | (0.387,0.429) |
| **222112** | 0.562 | (0.539,0.586) |
| **322112** | 0.640 | (0.618,0.663) |
| **132112** | 0.499 | (0.479,0.519) |
| **232112** | 0.653 | (0.630,0.677) |
| **332112** | 0.731 | (0.709,0.754) |
| **113112** | 0.340 | (0.322,0.357) |
| **213112** | 0.494 | (0.475,0.514) |
| **313112** | 0.571 | (0.553,0.592) |
| **123112** | 0.447 | (0.427,0.468) |
| **223112** | 0.601 | (0.580,0.624) |
| **323112** | 0.679 | (0.658,0.701) |
| **133112** | 0.538 | (0.519,0.557) |
| **233112** | 0.692 | (0.671,0.715) |
| **333112** | 0.770 | (0.749,0.792) |
| **111212** | 0.252 | (0.231,0.272) |
| **211212** | 0.405 | (0.383,0.428) |
| **311212** | 0.483 | (0.462,0.505) |
| **121212** | 0.359 | (0.337,0.381) |
| **221212** | 0.513 | (0.488,0.538) |
| **321212** | 0.591 | (0.568,0.615) |
| **131212** | 0.450 | (0.428,0.472) |
| **231212** | 0.604 | (0.579,0.629) |
| **331212** | 0.682 | (0.658,0.706) |
| **112212** | 0.373 | (0.353,0.394) |
| **212212** | 0.528 | (0.505,0.551) |
| **312212** | 0.605 | (0.583,0.628) |
| **122212** | 0.481 | (0.458,0.504) |
| **222212** | 0.635 | (0.609,0.662) |
| **322212** | 0.712 | (0.688,0.738) |
| **132212** | 0.572 | (0.549,0.594) |
| **232212** | 0.726 | (0.700,0.753) |
| **332212** | 0.803 | (0.779,0.830) |
| **113212** | 0.413 | (0.392,0.434) |
| **213212** | 0.567 | (0.545,0.590) |
| **313212** | 0.644 | (0.623,0.667) |
| **123212** | 0.520 | (0.498,0.543) |
| **223212** | 0.674 | (0.650,0.700) |
| **323212** | 0.752 | (0.728,0.777) |
| **133212** | 0.611 | (0.589,0.633) |
| **233212** | 0.765 | (0.741,0.791) |
| **333212** | 0.843 | (0.819,0.868) |
| **111312** | 0.272 | (0.253,0.291) |
| **211312** | 0.426 | (0.406,0.447) |
| **311312** | 0.504 | (0.484,0.524) |
| **121312** | 0.380 | (0.359,0.400) |
| **221312** | 0.534 | (0.512,0.556) |
| **321312** | 0.611 | (0.591,0.633) |
| **131312** | 0.471 | (0.451,0.490) |
| **231312** | 0.625 | (0.603,0.647) |
| **331312** | 0.702 | (0.681,0.724) |
| **112312** | 0.394 | (0.375,0.413) |
| **212312** | 0.548 | (0.527,0.569) |
| **312312** | 0.626 | (0.605,0.646) |
| **122312** | 0.501 | (0.480,0.522) |
| **222312** | 0.656 | (0.632,0.680) |
| **322312** | 0.733 | (0.711,0.757) |
| **132312** | 0.592 | (0.572,0.613) |
| **232312** | 0.747 | (0.723,0.771) |
| **332312** | 0.824 | (0.802,0.848) |
| **113312** | 0.433 | (0.415,0.452) |
| **213312** | 0.587 | (0.568,0.608) |
| **313312** | 0.665 | (0.646,0.685) |
| **123312** | 0.541 | (0.521,0.561) |
| **223312** | 0.695 | (0.673,0.718) |
| **323312** | 0.773 | (0.752,0.795) |
| **133312** | 0.632 | (0.613,0.651) |
| **233312** | 0.786 | (0.764,0.809) |
| **333312** | 0.864 | (0.842,0.886) |
| **111122** | 0.263 | (0.243,0.284) |
| **211122** | 0.417 | (0.395,0.440) |
| **311122** | 0.495 | (0.474,0.516) |
| **121122** | 0.371 | (0.349,0.393) |
| **221122** | 0.525 | (0.500,0.549) |
| **321122** | 0.602 | (0.579,0.626) |
| **131122** | 0.462 | (0.440,0.483) |
| **231122** | 0.616 | (0.591,0.641) |
| **331122** | 0.693 | (0.670,0.718) |
| **112122** | 0.385 | (0.364,0.406) |
| **212122** | 0.539 | (0.515,0.563) |
| **312122** | 0.617 | (0.594,0.640) |
| **122122** | 0.492 | (0.470,0.516) |
| **222122** | 0.647 | (0.620,0.673) |
| **322122** | 0.724 | (0.699,0.750) |
| **132122** | 0.583 | (0.561,0.607) |
| **232122** | 0.737 | (0.711,0.764) |
| **332122** | 0.815 | (0.790,0.842) |
| **113122** | 0.424 | (0.405,0.445) |
| **213122** | 0.578 | (0.557,0.601) |
| **313122** | 0.656 | (0.635,0.678) |
| **123122** | 0.532 | (0.510,0.555) |
| **223122** | 0.686 | (0.661,0.711) |
| **323122** | 0.764 | (0.740,0.788) |
| **133122** | 0.623 | (0.601,0.645) |
| **233122** | 0.777 | (0.752,0.802) |
| **333122** | 0.855 | (0.831,0.880) |
| **111222** | 0.336 | (0.314,0.359) |
| **211222** | 0.490 | (0.465,0.515) |
| **311222** | 0.568 | (0.544,0.592) |
| **121222** | 0.444 | (0.419,0.467) |
| **221222** | 0.598 | (0.571,0.625) |
| **321222** | 0.675 | (0.650,0.702) |
| **131222** | 0.535 | (0.511,0.559) |
| **231222** | 0.689 | (0.661,0.716) |
| **331222** | 0.766 | (0.741,0.793) |
| **112222** | 0.458 | (0.435,0.481) |
| **212222** | 0.612 | (0.586,0.638) |
| **312222** | 0.690 | (0.665,0.715) |
| **122222** | 0.565 | (0.541,0.590) |
| **222222** | 0.719 | (0.691,0.748) |
| **322222** | 0.797 | (0.770,0.825) |
| **132222** | 0.656 | (0.632,0.681) |
| **232222** | 0.810 | (0.782,0.840) |
| **332222** | 0.888 | (0.861,0.916) |
| **113222** | 0.497 | (0.476,0.520) |
| **213222** | 0.651 | (0.627,0.677) |
| **313222** | 0.729 | (0.706,0.754) |
| **123222** | 0.605 | (0.581,0.629) |
| **223222** | 0.759 | (0.732,0.787) |
| **323222** | 0.837 | (0.811,0.864) |
| **133222** | 0.696 | (0.672,0.720) |
| **233222** | 0.850 | (0.823,0.878) |
| **333222** | 0.927 | (0.902,0.955) |
| **111322** | 0.357 | (0.335,0.379) |
| **211322** | 0.511 | (0.488,0.534) |
| **311322** | 0.588 | (0.567,0.611) |
| **121322** | 0.464 | (0.441,0.487) |
| **221322** | 0.618 | (0.593,0.644) |
| **321322** | 0.696 | (0.672,0.721) |
| **131322** | 0.555 | (0.533,0.578) |
| **231322** | 0.709 | (0.684,0.735) |
| **331322** | 0.787 | (0.763,0.812) |
| **112322** | 0.479 | (0.456,0.501) |
| **212322** | 0.633 | (0.608,0.657) |
| **312322** | 0.710 | (0.687,0.734) |
| **122322** | 0.586 | (0.562,0.610) |
| **222322** | 0.740 | (0.713,0.767) |
| **322322** | 0.818 | (0.792,0.844) |
| **132322** | 0.677 | (0.654,0.701) |
| **232322** | 0.831 | (0.804,0.859) |
| **332322** | 0.909 | (0.883,0.935) |
| **113322** | 0.518 | (0.497,0.539) |
| **213322** | 0.672 | (0.650,0.695) |
| **313322** | 0.749 | (0.728,0.773) |
| **123322** | 0.625 | (0.603,0.648) |
| **223322** | 0.780 | (0.755,0.805) |
| **323322** | 0.857 | (0.834,0.882) |
| **133322** | 0.716 | (0.695,0.739) |
| **233322** | 0.871 | (0.846,0.896) |
| **333322** | 0.948 | (0.925,0.973) |
| **111132** | 0.295 | (0.275,0.314) |
| **211132** | 0.449 | (0.428,0.470) |
| **311132** | 0.526 | (0.506,0.547) |
| **121132** | 0.402 | (0.381,0.423) |
| **221132** | 0.556 | (0.533,0.579) |
| **321132** | 0.634 | (0.612,0.656) |
| **131132** | 0.493 | (0.473,0.514) |
| **231132** | 0.647 | (0.624,0.670) |
| **331132** | 0.725 | (0.703,0.748) |
| **112132** | 0.416 | (0.396,0.436) |
| **212132** | 0.570 | (0.548,0.593) |
| **312132** | 0.648 | (0.627,0.670) |
| **122132** | 0.524 | (0.503,0.546) |
| **222132** | 0.678 | (0.654,0.703) |
| **322132** | 0.756 | (0.732,0.780) |
| **132132** | 0.615 | (0.594,0.636) |
| **232132** | 0.769 | (0.744,0.794) |
| **332132** | 0.847 | (0.823,0.871) |
| **113132** | 0.456 | (0.437,0.475) |
| **213132** | 0.610 | (0.589,0.631) |
| **313132** | 0.688 | (0.668,0.708) |
| **123132** | 0.563 | (0.543,0.585) |
| **223132** | 0.717 | (0.695,0.742) |
| **323132** | 0.795 | (0.773,0.818) |
| **133132** | 0.654 | (0.634,0.675) |
| **233132** | 0.808 | (0.786,0.832) |
| **333132** | 0.886 | (0.864,0.909) |
| **111232** | 0.367 | (0.346,0.390) |
| **211232** | 0.522 | (0.498,0.547) |
| **311232** | 0.599 | (0.576,0.623) |
| **121232** | 0.475 | (0.452,0.498) |
| **221232** | 0.629 | (0.603,0.655) |
| **321232** | 0.707 | (0.682,0.732) |
| **131232** | 0.566 | (0.543,0.589) |
| **231232** | 0.720 | (0.694,0.746) |
| **331232** | 0.797 | (0.773,0.824) |
| **112232** | 0.489 | (0.468,0.511) |
| **212232** | 0.643 | (0.619,0.668) |
| **312232** | 0.721 | (0.698,0.745) |
| **122232** | 0.597 | (0.574,0.621) |
| **222232** | 0.751 | (0.724,0.778) |
| **322232** | 0.829 | (0.803,0.855) |
| **132232** | 0.688 | (0.665,0.712) |
| **232232** | 0.842 | (0.815,0.870) |
| **332232** | 0.919 | (0.894,0.946) |
| **113232** | 0.529 | (0.508,0.551) |
| **213232** | 0.683 | (0.659,0.708) |
| **313232** | 0.760 | (0.738,0.785) |
| **123232** | 0.636 | (0.613,0.660) |
| **223232** | 0.791 | (0.765,0.817) |
| **323232** | 0.868 | (0.843,0.894) |
| **133232** | 0.727 | (0.705,0.751) |
| **233232** | 0.881 | (0.856,0.908) |
| **333232** | 0.959 | (0.934,0.985) |
| **111332** | 0.388 | (0.367,0.409) |
| **211332** | 0.542 | (0.520,0.565) |
| **311332** | 0.620 | (0.599,0.642) |
| **121332** | 0.496 | (0.474,0.517) |
| **221332** | 0.650 | (0.626,0.674) |
| **321332** | 0.727 | (0.705,0.751) |
| **131332** | 0.587 | (0.566,0.608) |
| **231332** | 0.741 | (0.717,0.765) |
| **331332** | 0.818 | (0.796,0.842) |
| **112332** | 0.510 | (0.490,0.530) |
| **212332** | 0.664 | (0.642,0.687) |
| **312332** | 0.742 | (0.720,0.764) |
| **122332** | 0.617 | (0.596,0.640) |
| **222332** | 0.771 | (0.747,0.797) |
| **322332** | 0.849 | (0.825,0.873) |
| **132332** | 0.708 | (0.687,0.731) |
| **232332** | 0.862 | (0.838,0.888) |
| **332332** | 0.940 | (0.917,0.965) |
| **113332** | 0.549 | (0.530,0.569) |
| **213332** | 0.703 | (0.683,0.726) |
| **313332** | 0.781 | (0.761,0.803) |
| **123332** | 0.657 | (0.636,0.678) |
| **223332** | 0.811 | (0.788,0.835) |
| **323332** | 0.889 | (0.867,0.912) |
| **133332** | 0.748 | (0.728,0.769) |
| **233332** | 0.902 | (0.879,0.925) |
| **333332** | 0.980 | (0.958,1.003) |
| **111113** | 0.200 | (0.183,0.216) |
| **211113** | 0.354 | (0.335,0.372) |
| **311113** | 0.432 | (0.414,0.449) |
| **121113** | 0.307 | (0.289,0.326) |
| **221113** | 0.461 | (0.442,0.482) |
| **321113** | 0.539 | (0.520,0.559) |
| **131113** | 0.398 | (0.381,0.416) |
| **231113** | 0.552 | (0.533,0.572) |
| **331113** | 0.630 | (0.611,0.649) |
| **112113** | 0.321 | (0.303,0.340) |
| **212113** | 0.476 | (0.455,0.496) |
| **312113** | 0.553 | (0.534,0.574) |
| **122113** | 0.429 | (0.409,0.450) |
| **222113** | 0.583 | (0.561,0.606) |
| **322113** | 0.661 | (0.640,0.684) |
| **132113** | 0.520 | (0.501,0.540) |
| **232113** | 0.674 | (0.652,0.697) |
| **332113** | 0.752 | (0.730,0.775) |
| **113113** | 0.361 | (0.343,0.379) |
| **213113** | 0.515 | (0.496,0.535) |
| **313113** | 0.593 | (0.574,0.612) |
| **123113** | 0.469 | (0.450,0.488) |
| **223113** | 0.622 | (0.602,0.644) |
| **323113** | 0.700 | (0.680,0.721) |
| **133113** | 0.560 | (0.541,0.578) |
| **233113** | 0.714 | (0.693,0.735) |
| **333113** | 0.791 | (0.771,0.812) |
| **111213** | 0.273 | (0.254,0.291) |
| **211213** | 0.427 | (0.406,0.447) |
| **311213** | 0.505 | (0.485,0.524) |
| **121213** | 0.380 | (0.360,0.401) |
| **221213** | 0.534 | (0.512,0.557) |
| **321213** | 0.612 | (0.591,0.634) |
| **131213** | 0.471 | (0.452,0.491) |
| **231213** | 0.625 | (0.603,0.648) |
| **331213** | 0.703 | (0.682,0.724) |
| **112213** | 0.394 | (0.375,0.415) |
| **212213** | 0.549 | (0.526,0.571) |
| **312213** | 0.626 | (0.605,0.648) |
| **122213** | 0.502 | (0.480,0.524) |
| **222213** | 0.656 | (0.631,0.681) |
| **322213** | 0.734 | (0.711,0.758) |
| **132213** | 0.593 | (0.572,0.615) |
| **232213** | 0.747 | (0.723,0.772) |
| **332213** | 0.824 | (0.802,0.849) |
| **113213** | 0.434 | (0.415,0.454) |
| **213213** | 0.588 | (0.567,0.610) |
| **313213** | 0.666 | (0.645,0.687) |
| **123213** | 0.542 | (0.520,0.563) |
| **223213** | 0.696 | (0.672,0.720) |
| **323213** | 0.773 | (0.751,0.796) |
| **133213** | 0.633 | (0.612,0.653) |
| **233213** | 0.787 | (0.763,0.810) |
| **333213** | 0.864 | (0.842,0.887) |
| **111313** | 0.293 | (0.276,0.311) |
| **211313** | 0.448 | (0.428,0.466) |
| **311313** | 0.525 | (0.507,0.543) |
| **121313** | 0.401 | (0.382,0.419) |
| **221313** | 0.555 | (0.535,0.575) |
| **321313** | 0.633 | (0.614,0.652) |
| **131313** | 0.492 | (0.474,0.510) |
| **231313** | 0.646 | (0.626,0.666) |
| **331313** | 0.724 | (0.705,0.743) |
| **112313** | 0.415 | (0.396,0.434) |
| **212313** | 0.569 | (0.548,0.590) |
| **312313** | 0.647 | (0.627,0.667) |
| **122313** | 0.523 | (0.502,0.543) |
| **222313** | 0.677 | (0.654,0.700) |
| **322313** | 0.754 | (0.733,0.777) |
| **132313** | 0.614 | (0.594,0.633) |
| **232313** | 0.768 | (0.745,0.791) |
| **332313** | 0.845 | (0.824,0.868) |
| **113313** | 0.455 | (0.437,0.472) |
| **213313** | 0.609 | (0.590,0.628) |
| **313313** | 0.686 | (0.668,0.705) |
| **123313** | 0.562 | (0.543,0.581) |
| **223313** | 0.716 | (0.696,0.737) |
| **323313** | 0.794 | (0.774,0.814) |
| **133313** | 0.653 | (0.635,0.671) |
| **233313** | 0.807 | (0.786,0.828) |
| **333313** | 0.885 | (0.865,0.905) |
| **111123** | 0.285 | (0.265,0.303) |
| **211123** | 0.439 | (0.418,0.459) |
| **311123** | 0.516 | (0.497,0.536) |
| **121123** | 0.392 | (0.372,0.412) |
| **221123** | 0.546 | (0.524,0.569) |
| **321123** | 0.624 | (0.602,0.645) |
| **131123** | 0.483 | (0.463,0.503) |
| **231123** | 0.637 | (0.615,0.660) |
| **331123** | 0.715 | (0.694,0.737) |
| **112123** | 0.406 | (0.386,0.426) |
| **212123** | 0.560 | (0.537,0.583) |
| **312123** | 0.638 | (0.616,0.660) |
| **122123** | 0.514 | (0.492,0.536) |
| **222123** | 0.667 | (0.643,0.693) |
| **322123** | 0.745 | (0.722,0.770) |
| **132123** | 0.605 | (0.583,0.627) |
| **232123** | 0.759 | (0.734,0.784) |
| **332123** | 0.836 | (0.813,0.861) |
| **113123** | 0.445 | (0.426,0.465) |
| **213123** | 0.600 | (0.579,0.621) |
| **313123** | 0.678 | (0.657,0.698) |
| **123123** | 0.553 | (0.532,0.574) |
| **223123** | 0.707 | (0.684,0.731) |
| **323123** | 0.785 | (0.763,0.807) |
| **133123** | 0.644 | (0.624,0.665) |
| **233123** | 0.798 | (0.775,0.822) |
| **333123** | 0.876 | (0.854,0.898) |
| **111223** | 0.357 | (0.337,0.378) |
| **211223** | 0.511 | (0.489,0.534) |
| **311223** | 0.589 | (0.568,0.610) |
| **121223** | 0.465 | (0.443,0.487) |
| **221223** | 0.619 | (0.595,0.644) |
| **321223** | 0.696 | (0.674,0.720) |
| **131223** | 0.556 | (0.535,0.577) |
| **231223** | 0.710 | (0.685,0.735) |
| **331223** | 0.788 | (0.765,0.811) |
| **112223** | 0.479 | (0.457,0.501) |
| **212223** | 0.633 | (0.609,0.657) |
| **312223** | 0.711 | (0.688,0.734) |
| **122223** | 0.586 | (0.564,0.610) |
| **222223** | 0.741 | (0.714,0.767) |
| **322223** | 0.818 | (0.794,0.844) |
| **132223** | 0.677 | (0.655,0.701) |
| **232223** | 0.832 | (0.805,0.859) |
| **332223** | 0.909 | (0.884,0.935) |
| **113223** | 0.519 | (0.498,0.540) |
| **213223** | 0.673 | (0.650,0.696) |
| **313223** | 0.750 | (0.729,0.773) |
| **123223** | 0.626 | (0.604,0.648) |
| **223223** | 0.780 | (0.755,0.806) |
| **323223** | 0.858 | (0.835,0.882) |
| **133223** | 0.717 | (0.696,0.739) |
| **233223** | 0.871 | (0.846,0.897) |
| **333223** | 0.949 | (0.926,0.973) |
| **111323** | 0.378 | (0.358,0.397) |
| **211323** | 0.532 | (0.510,0.553) |
| **311323** | 0.610 | (0.590,0.630) |
| **121323** | 0.485 | (0.465,0.506) |
| **221323** | 0.639 | (0.617,0.663) |
| **321323** | 0.717 | (0.696,0.739) |
| **131323** | 0.576 | (0.556,0.597) |
| **231323** | 0.730 | (0.708,0.753) |
| **331323** | 0.808 | (0.787,0.830) |
| **112323** | 0.500 | (0.478,0.521) |
| **212323** | 0.654 | (0.630,0.677) |
| **312323** | 0.731 | (0.709,0.754) |
| **122323** | 0.607 | (0.585,0.630) |
| **222323** | 0.761 | (0.737,0.787) |
| **322323** | 0.839 | (0.815,0.864) |
| **132323** | 0.698 | (0.677,0.720) |
| **232323** | 0.852 | (0.827,0.878) |
| **332323** | 0.930 | (0.907,0.954) |
| **113323** | 0.539 | (0.520,0.559) |
| **213323** | 0.693 | (0.672,0.714) |
| **313323** | 0.771 | (0.751,0.792) |
| **123323** | 0.647 | (0.626,0.668) |
| **223323** | 0.801 | (0.778,0.824) |
| **323323** | 0.878 | (0.857,0.901) |
| **133323** | 0.738 | (0.718,0.758) |
| **233323** | 0.892 | (0.869,0.915) |
| **333323** | 0.969 | (0.948,0.992) |
| **111133** | 0.316 | (0.298,0.333) |
| **211133** | 0.470 | (0.451,0.488) |
| **311133** | 0.548 | (0.529,0.566) |
| **121133** | 0.423 | (0.404,0.442) |
| **221133** | 0.577 | (0.557,0.599) |
| **321133** | 0.655 | (0.636,0.675) |
| **131133** | 0.514 | (0.496,0.533) |
| **231133** | 0.668 | (0.648,0.689) |
| **331133** | 0.746 | (0.727,0.766) |
| **112133** | 0.438 | (0.418,0.456) |
| **212133** | 0.592 | (0.570,0.613) |
| **312133** | 0.669 | (0.649,0.689) |
| **122133** | 0.545 | (0.525,0.566) |
| **222133** | 0.699 | (0.676,0.722) |
| **322133** | 0.777 | (0.755,0.799) |
| **132133** | 0.636 | (0.616,0.656) |
| **232133** | 0.790 | (0.767,0.813) |
| **332133** | 0.868 | (0.846,0.890) |
| **113133** | 0.477 | (0.459,0.495) |
| **213133** | 0.631 | (0.612,0.651) |
| **313133** | 0.709 | (0.690,0.728) |
| **123133** | 0.585 | (0.565,0.604) |
| **223133** | 0.739 | (0.718,0.760) |
| **323133** | 0.816 | (0.796,0.837) |
| **133133** | 0.676 | (0.657,0.694) |
| **233133** | 0.830 | (0.809,0.852) |
| **333133** | 0.907 | (0.887,0.928) |
| **111233** | 0.389 | (0.369,0.408) |
| **211233** | 0.543 | (0.522,0.565) |
| **311233** | 0.620 | (0.600,0.641) |
| **121233** | 0.496 | (0.476,0.517) |
| **221233** | 0.650 | (0.627,0.673) |
| **321233** | 0.728 | (0.707,0.750) |
| **131233** | 0.587 | (0.568,0.608) |
| **231233** | 0.741 | (0.719,0.765) |
| **331233** | 0.819 | (0.798,0.841) |
| **112233** | 0.510 | (0.490,0.531) |
| **212233** | 0.665 | (0.642,0.688) |
| **312233** | 0.742 | (0.720,0.764) |
| **122233** | 0.618 | (0.596,0.640) |
| **222233** | 0.772 | (0.747,0.797) |
| **322233** | 0.850 | (0.827,0.874) |
| **132233** | 0.709 | (0.688,0.731) |
| **232233** | 0.863 | (0.838,0.888) |
| **332233** | 0.941 | (0.917,0.965) |
| **113233** | 0.550 | (0.530,0.570) |
| **213233** | 0.704 | (0.682,0.727) |
| **313233** | 0.782 | (0.761,0.803) |
| **123233** | 0.657 | (0.636,0.679) |
| **223233** | 0.812 | (0.788,0.836) |
| **323233** | 0.889 | (0.867,0.912) |
| **133233** | 0.749 | (0.728,0.769) |
| **233233** | 0.903 | (0.879,0.927) |
| **333233** | 0.980 | (0.959,1.003) |
| **111333** | 0.409 | (0.391,0.428) |
| **211333** | 0.563 | (0.544,0.584) |
| **311333** | 0.641 | (0.623,0.660) |
| **121333** | 0.517 | (0.498,0.535) |
| **221333** | 0.671 | (0.651,0.693) |
| **321333** | 0.749 | (0.729,0.768) |
| **131333** | 0.608 | (0.590,0.626) |
| **231333** | 0.762 | (0.741,0.783) |
| **331333** | 0.840 | (0.820,0.859) |
| **112333** | 0.531 | (0.512,0.550) |
| **212333** | 0.685 | (0.664,0.706) |
| **312333** | 0.763 | (0.742,0.783) |
| **122333** | 0.639 | (0.619,0.659) |
| **222333** | 0.793 | (0.770,0.816) |
| **322333** | 0.870 | (0.849,0.892) |
| **132333** | 0.730 | (0.710,0.750) |
| **232333** | 0.884 | (0.861,0.906) |
| **332333** | 0.961 | (0.940,0.983) |
| **113333** | 0.571 | (0.553,0.589) |
| **213333** | 0.725 | (0.706,0.744) |
| **313333** | 0.802 | (0.784,0.821) |
| **123333** | 0.678 | (0.659,0.697) |
| **223333** | 0.832 | (0.812,0.853) |
| **323333** | 0.910 | (0.891,0.930) |
| **133333** | 0.769 | (0.752,0.787) |
| **233333** | 0.923 | (0.903,0.944) |
| **333333** | 1.001 | (0.982,1.021) |

Table S9. Value set of weights for each CALY-SWE state, with 95% credible interval. Ordered from lowest capability state (111111) to highest capability state (333333).
